# UVC light modulates vitamin C and phenolic biosynthesis in acerola fruit: role of increased mitochondria activity and ROS production

**DOI:** 10.1038/s41598-020-78948-1

**Published:** 2020-12-15

**Authors:** Marcela Cristina Rabelo, Woo Young Bang, Vimal Nair, Ricardo Elesbao Alves, Daniel A. Jacobo-Velázquez, Shareena Sreedharan, Maria Raquel Alcântara de Miranda, Luis Cisneros-Zevallos

**Affiliations:** 1 Department of Horticultural Sciences, Texas A&M University, College Station, TX 77843 USA; 2 Department of Biochemistry and Molecular Biology, Universidade Federal do Ceará, Fortaleza, CE Brazil; 3 National Institute of Biological Resources (NIBR), Environmental Research Complex, Incheon, 404-708 Republic of Korea; 4 Embrapa Tropical Agroindustry, Fortaleza, CE Brazil; 5 Tecnologico de Monterrey, Escuela de Ingeniería y Ciencias, Ave. Gral Ramón Corona No. 2514. Colonia Nuevo México, CP 45201 Zapopan, Jal México

**Keywords:** Abiotic, Secondary metabolism

## Abstract

The effects of ultraviolet-C light (UVC) on vitamin C and phenolic compounds in acerola during postharvest storage were investigated in order to elucidate the mechanism inducing the antioxidant systems. The fruits, stored at 10 °C for 7 days after a hormetic UVC irradiation (two pulses of 0.3 J/cm^2^), showed significantly less degradation of vitamin C and phenolic compounds than the control without the UVC challenge. UVC activated the L-galactono-1,4-lactone dehydrogenase (GalDH), a key enzyme for vitamin C biosynthesis, and altered the composition of phenolic compounds, through phenolic biosynthesis, in acerola during postharvest storage. UVC also induced reactive oxygen species (ROS) productions at immediate (day 0) and late (day 7) times during postharvest storage through the mitochondrial electron transport chain and NADPH oxidase, respectively. Results suggest that UVC helps in the retention of vitamin C and phenolic content in acerola by altering ascorbic acid and phenolic metabolism through an increase in mitochondrial activity and a ROS-mediated mechanism. Data showed the beneficial effects of UVC on maintenance of nutraceutical quality in acerola during postharvest storage and supplied new insights into understanding the mechanism by which UVC irradiation enhance the antioxidant system in fruits.

## Introduction

Consumption of tropical fruits has increased significantly in the past few years due to their attractive taste and nutritional value, which includes antioxidants such as vitamins and phenolic compounds. However, these fruits are highly perishable with limited shelf-life due to qualitative and quantitative deterioration during postharvest storage, which is mediated by a rapid maturation and senescence process^1^. To preserve their quality during storage, several postharvest techniques have been developed to delay senescence, among which ultraviolet (UV) irradiation, has received much attention due to its effects on surface microbial growth, produce shelf-life extension and induction of the biosynthesis of phenolic antioxidants.

The main hydrosoluble antioxidants present in fruits are vitamin C (L-ascorbic acid) and phenolic compounds^2^. Vitamin C (L-ascorbic acid) is synthesized from D-glucose in plants by enzymes including GDP-D-mannose pyrophosphorylase (GMP), GDP-mannose 3′,5′-epimerase (GME), GDP-L-galactose phosphorylase (GGP), L-galactose dehydrogenase (GDH) and L-galactono-1,4-lactone dehydrogenase (GalDH)^3^. On the other hand, phenolic compounds are synthesized via the phenylpropanoid metabolism, which key enzyme is the phenylalanine ammonia lyase (PAL)^4,5^.

Acerola (*Malpighia emarginata* D.C.) is a tropical fruit, well known as a healthy fruit due to its high content of nutraceutical compounds like phenolics and vitamin C^6^, making acerola a good model to study the metabolism of those compounds. In addition, acerola is a highly perishable fruit with rapid loss of the nutraceutical quality during its postharvest life^7^. There is a need for the use of postharvest techniques like UVC that could preserve quality and extent its postharvest shelf-life.

UVC light (200–280 nm), which is part of the UV region (100–400 nm) is an abiotic stress with germicidal effect on microorganisms such as bacteria, yeasts, molds and viruses^8^. It is considered a cost effective non-thermal emerging technology and recent studies show a potential to ensure microbial safety of vegetable products and maintain their nutritional characteristics^9^. As a postharvest treatment, UVC irradiation at low doses extend the postharvest life and preserve the quality of tropical fruits by delaying fruit ripening and senescence, whereas the irradiation at high doses induces detrimental effects^10,11^. UVC irradiation increases the antioxidant activity and nutraceutical content of fruits, associated to an increase in phenols and flavonoids in guava and banana during storage after a 30 min UVC exposure^12,13^. UVC significantly reduced decay in strawberry stored at 10 °C by increasing the activity of antioxidant enzymes and non-enzymatic antioxidants glutathione (GSH) and phenolic compounds^14^. UVC radiation also reduced decay due to over ripening in blueberries by enhancing the antioxidant levels^15^. Significant increases in flavonoids and total phenolic contents were found in mandarin fruit treated with 1.5 and 3.0 kJ/m^2^ UVC and stored for 3 days^16^, similar results were reported for papaya^17^. These data suggest that sub-lethal doses of UVC mediate the induction of antioxidant systems as secondary response to environmental stress, resulting in the extension of postharvest life and quality of a range of fruits. However, the mechanism by which UVC irradiation enhances the antioxidant level in fruits remains unknown.

It has been hypothesized that ROS production induced by UVC may mediate the enhancement of the antioxidant potential^18,19^. The superoxide radical (O_2_·^−^) as a primary ROS triggers a chemical reaction cascade leading to the formation of various ROS and induces antioxidant compounds like polyphenols through MYB transcription factors^20^. Nevertheless, it remains unknown if UVC enhances the antioxidant enzymes such as SOD, CAT, POD, APX and GR through a ROS mediated mechanism in fruits^21^.

It has been reported that a sudden increase in intracellular ROS level is attributed to disturbances in the equilibrium between the production and the scavenging of ROS, affected by various abiotic stresses including wounding, salinity, UV radiation, drought, heavy metals, temperature extremes, nutrient deficiency and air pollution^22–24^. For plant cell adaptation to the abiotic stresses, mitochondria plays a central role in controlling ROS generation by means of energy-dissipating systems^24,25^. The mitochondrial electron transport chain (ETC) possesses electrons with sufficient free energy to directly reduce O_2_, which is considered as a primary source of mitochondrial ROS generation, and the complex I and III of mitochondrial ETC especially are the very well-known sites of O_2_·^−^ production^24^. If the ETC is under excess energy by the UVC irradiation, harboring photons with high energy as described above, may lead to the sudden ROS generation through the complex I and III of ETC. A sudden ROS generation can reduce the chances of large-scale ROS production at later time after the stress challenge by dissipating mitochondrial membrane potential (Δ*Ψ*), which have been demonstrated in previous studies using wheat mitochondria^26,27^.

Herein we investigated the use of UVC applied to acerola fruit and its effects on vitamin C and associated biosynthetic genes, phenolic metabolites and enzymes associated to their metabolism. Here we propose the mode of action by which UVC modulates levels of vitamin C and polyphenols in acerola fruit through an increased mitochondrial activity and production of reactive oxygen species (ROS). Our data supplies new insights into understanding the mechanism by which UVC irradiation enhances the antioxidant content in fruits.

## Results

### UVC effect on vitamin C content and enzymatic synthesis

**Figure 1:**
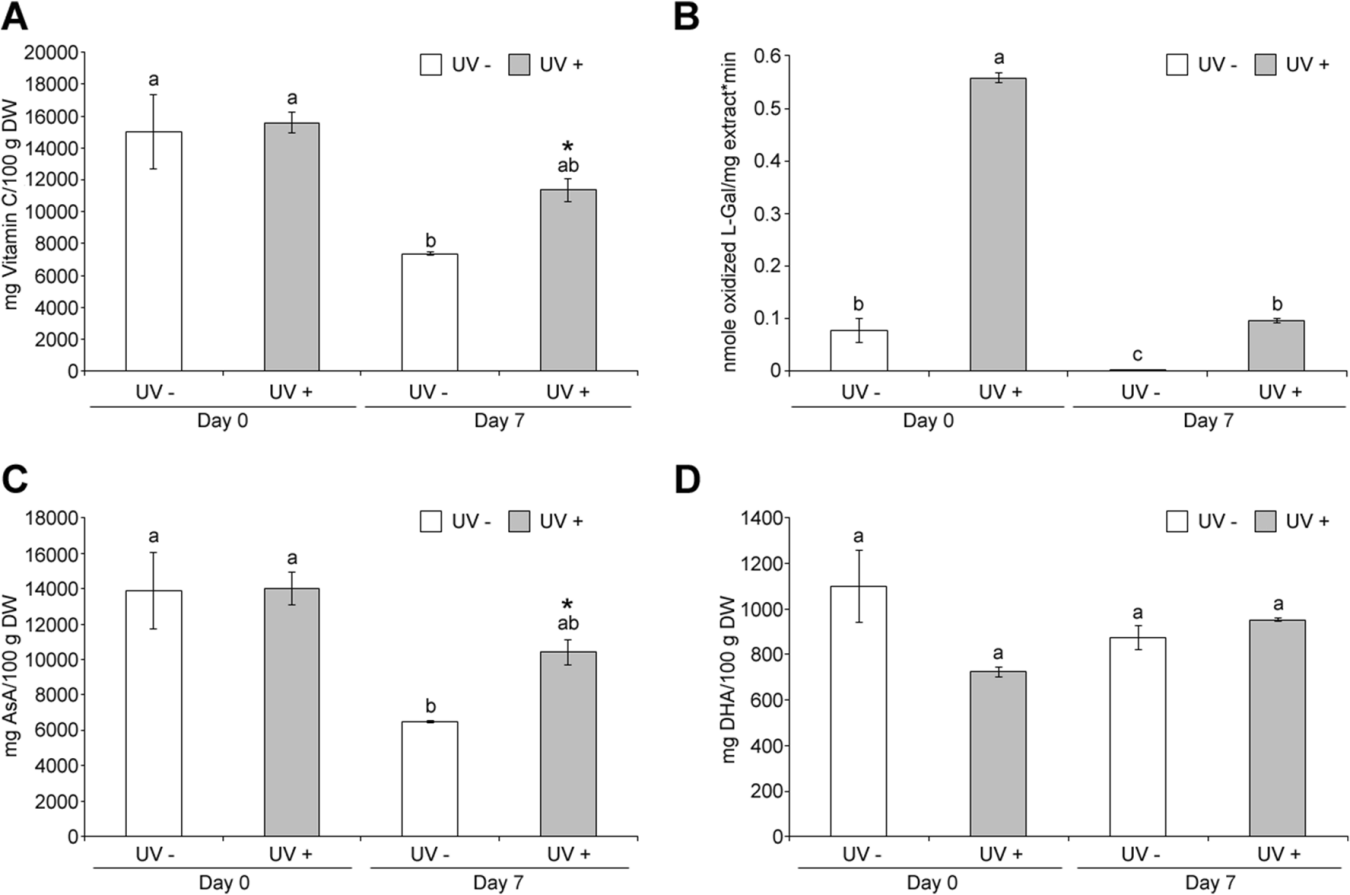
Effect of UVC on vitamin C metabolism. Acerola fruits without (UV−) or with UVC treatment (UV+), were stored at 10 °C for 0 day (Day 0) or 7 days (Day 7) and subjected to quantification of total vitamin C (**A**), AsA (**C**), DHA (**D**), and GalDH activity assay (**B**). Data, obtained from at least three biological repeats, are shown as mean ± SE values. Different letters indicate significant differences by the ANOVA/Tukey-HSD (*p* < 0.05) while asterisks in (**A**) and (**C**) represent significant differences between UVC treated (UV−) and untreated (UV+) samples at only Day 7, using the Student’s t-test (*p* < 0.05).

The initial vitamin C content (~ 15,000 mg/100 g DW) decreased during storage in acerola fruit. However, the effects of UVC irradiation on vitamin C metabolism showed that total vitamin C was significantly higher after 7 days of storage (~ 54%), when compared to control (Fig. 1a). As vitamin C predominantly exists in its reduced form, ascorbate (AsA), acerolas were evaluated for its reduced and oxidized form, dehydroascorbate (DHA) (Fig. 1c,d). After storage, UVC treated samples showed a significantly higher AsA content (~ 37%) than control (Fig. 1c), however both control and UVC treated samples showed no significant difference in DHA content (Fig. 1d). These results indicate that UVC helps in the retention of vitamin C, especially reduced AsA, from degradation during postharvest storage of acerola.

The L-galactono-1,4-lactone dehydrogenase (GalDH) a key enzyme producing vitamin C catalyzes the oxidation of L-galactono-1,4 lactone into ascorbic acid, the last step of ascorbic acid synthesis^3^. As shown in Fig. 1b, the GalDH activity decreased drastically after 7 days of storage in control fruits, however UVC treatment induced GalDH activity immediately at day 0 after irradiation, which was maintained even 7 days after treatment above the control levels. Previous studies have shown the increase in AsA by other stresses including methyl jasmonate^28^ and activation of GalDH by wounding^29^.

### UVC effects on expression of genes of vitamin C biosynthesis

**Figure 2:**
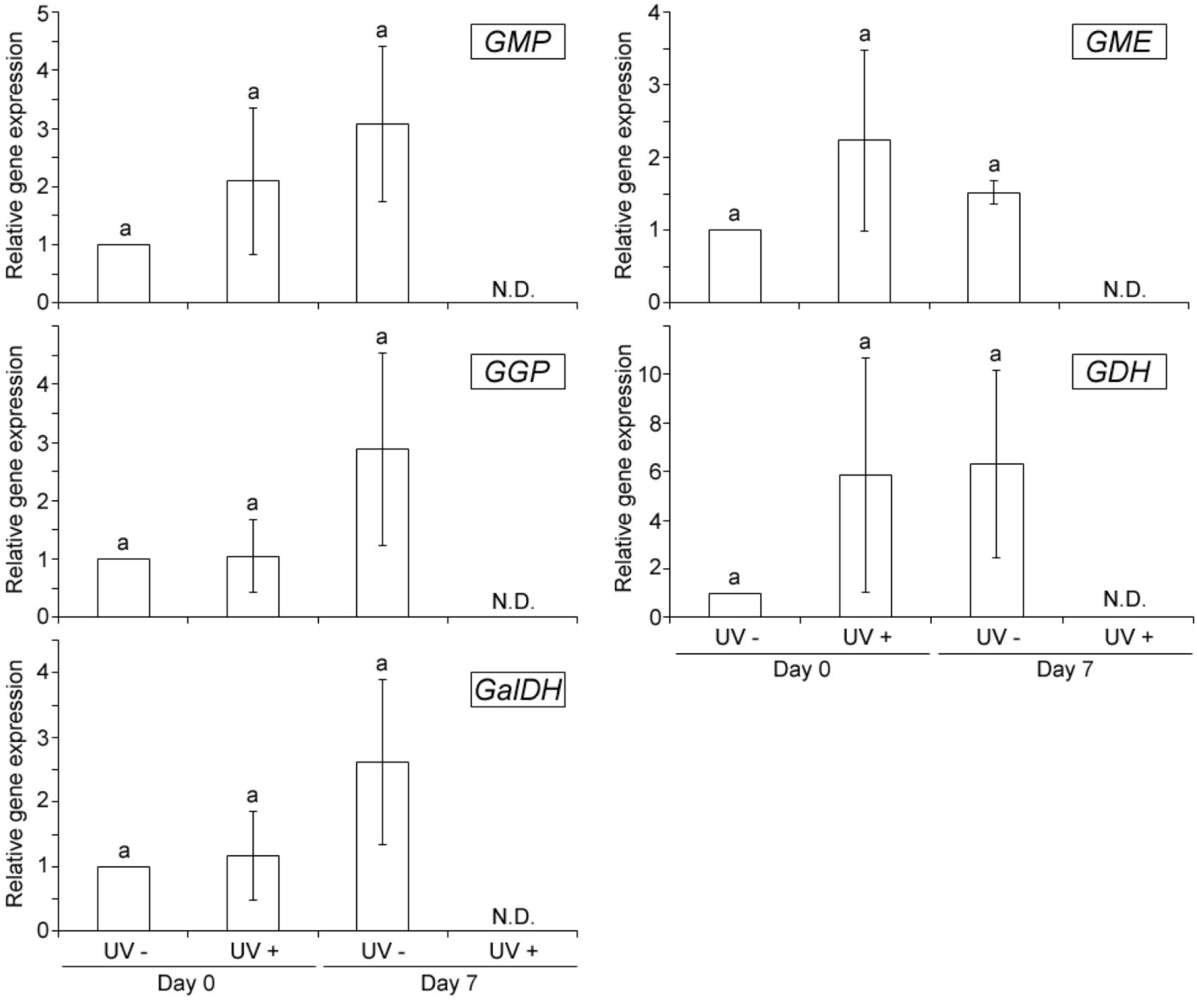
Effects of UVC on the expression of genes related to vitamin C metabolism. Acerola fruits without (UV−) or with UVC treatment (UV+), were stored at 10 °C for 0 day (Day 0) or 7 days (Day 7). Not detectable (ND), likely caused by oxidation of RNAs from the fruits. Data, obtained from triplicate repeats, are shown as mean ± SE values. Different letters indicate significant differences by the ANOVA/Tukey-HSD (*p* < 0.05).

As shown in Fig. 2, the UVC irradiation showed no significant effects on the gene expressions responsible for GDP-D-mannose pyrophosphorylase (GMP), GDP-mannose 3′,5′-epimerase (GME), GDP-L-galactose phosphorylase (GGP), L-galactose dehydrogenase (GDH) and GalDH enzymes, involved in the vitamin C metabolism^3^. Particularly, RNA solution, prepared from fruits at 7 days after the UVC treatment, showed brown color resulting in that any fluorescent signals, corresponding to PCR amplification, by all primer sets used were not detectable (N.D.), which is assumed to be caused by oxidation of RNAs from the fruits^30–32^ (Fig. 2).

### UVC effect on content and composition of phenolics

**Figure 3:**
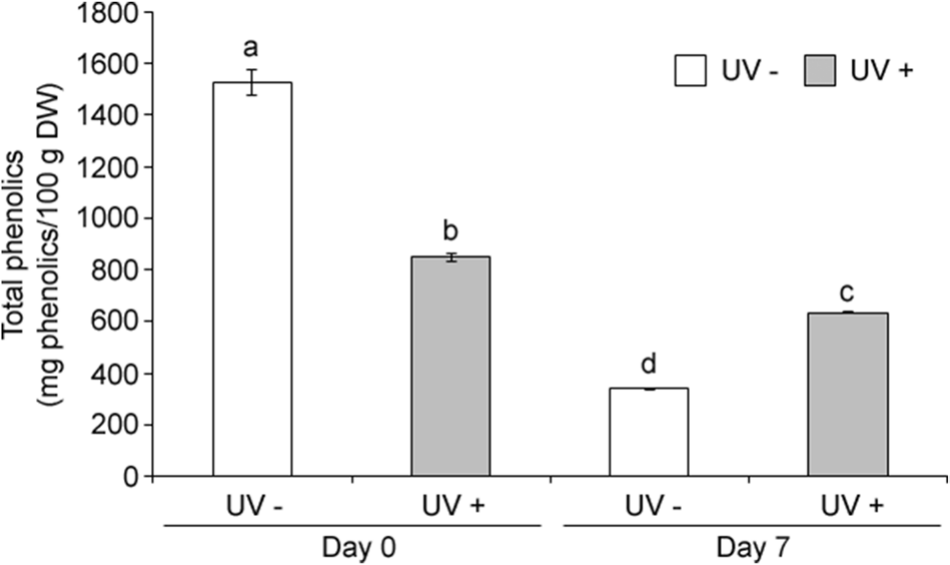
Effect of UVC on the content of total phenolic compounds. Acerola fruits without (UV−) or with UVC treatment (UV+), were stored at 10 °C for 0 day (Day 0) or 7 days (Day 7) and subjected to quantification of total phenolic compounds by LC–MS analysis (Table 1). Data, obtained from at least three biological repeats, are shown as mean ± SE values. Different letters indicate significant differences by the ANOVA/Tukey-HSD (*p* < 0.05).

The effects of UVC on total phenolic content in acerolas during postharvest storage were investigated (Fig. 3). Results showed that total phenolic content decreased in acerola fruits right after the UVC treatment by ~ 44%. However, total phenolic content decreased significantly less in UVC treated samples compared to control fruits after 7 days (Fig. 3) showing ~ 82% more phenolics than controls. These results indicate that UVC enhances the retention of phenolics in acerolas during postharvest storage.

Crude extracts were separated into four fractions and liquid chromatograms revealed that UV treated and control samples showed several peaks in F3 and F4 fractions with similar chromatograms (Fig. S1). Peaks were identified by mass spectrometry in F3 as phenolic acids (peaks 1c, 1d), kaempferol derivatives (peaks 6, 10, 11), isorhamnetin and derivatives (peaks 7, 8, 9), quercetin glycosides (peak 12, 13) and myricetin derivatives (peaks 14–16) while in F4 peaks were identified as cyanidin glycosides (peaks 1e, 5), pelargonidin derivatives (peaks 2, 3) and peonidin glycoside (peak 4) (Fig. S1, Table S1). The total amount of phenolics at day 0 was ~ 1526 mg/100 g DW in control samples containing ~ 0.45, 10.9 and 88.6% phenolic acids, flavonols and anthocyanins, respectively. The most abundant flavonols were peaks 8, 11, 15, 16 and for anthocyanins, peaks 1e and 2 (Fig. S1, Table 1). Details of peak identifications are presented as supplementary information.

**Table 1:** Effect of UVC light treatments on the content of phenolic acids and flavonoids from acerola fruit determined by LC–MS. Values represent concentration (mg phenolics/100 freeze-dried acerola).

Peak	Retention time (min)	Identification	Day 0		Day 7	
			UV−	UV+	UV−	UV+
1c	19.5–20.5	Gallic acid	2.9 a	1.3 d	2.3 b	1.5 c
1d	21.3–22.5	Coumaroyl quinic acid	4.0 c	5.3 b	3.9 c	8.1 a
1e	30.25	Cyanidin 3-rhamnoside	1152 a	416 b	47 d	276 c
2	33.03	Pelargonidin 3-rhamnoside	155 a	60 b	25 c	52 b
3	37.37	Pelargonidin	4 b	7 a	0.37 d	1.6 c
4	41.32	Peonidin-3-xylopyranoside	28 b	156 a	ND	6 c
5	46.19	Cyanidin 3-rutinoside	12 c	67 a	ND	19 b
6	47.04	Kaempferol 3-*O*-robinobioside-7-*O*-arabinofuranoside	3 c	1.47 d	5.5 a	3 b
7	53.18	Isorhamnetin 3-*O*-galactoside	3 a	3 a	ND	2.7 b
8	56.06	Isorhamnetin 3-O-glucoside	17 d	21 c	37 b	43 a
9	57.16	Isorhamnetin	1.2 b	1.1 c	1.3 a	ND
10	60.4	Kaempferol 7-*O*-neohesperidoside	5 b	3.3 c	3.1 d	7 a
11	65.88	Kaempferol 3-*O*-arabinofuranoside	10.5 d	13.8 c	17.6 b	18.1 a
12	67.19	Quercetin 3-*O*-glucoside	2.8 b	2.8 b	0.5 c	4.7 a
13	70.38	Quercitrin-2″-*O*-gallate	1.1 b	0.8 d	1.9 a	1.0 c
14	73.88	Myricetin-3-*O*-glucuronide	0.1 b	14.3 a	ND	ND
15	74.36	Myricetin-3-*O*-glucoside	16.1 b	0.1 c	0.25 c	25.1 a
16	76–77.85	Myricetin rhamnoside	107 c	74 d	196 a	167 b

Values represent the mean of three replicates. Values with different letters within the same compound represent statistical difference (*p* < 0.05) by the Tukey’s HSD test. Concentration values for each type of compound were determined from chromatogram peak areas and standards (Table S1). Areas of peaks 1e to 5 were quantified at 520 nm (Fraction F4) and areas of peaks 1c, 1d, 6–16 were quantified at 330 nm (Fraction F3).

*ND *not detected.

Based on the chromatograms there was a decrease on most flavonols on day 0 after the UVC treatment compared to controls (*p* < 0.05), however, at day 7 there was an increase in most peaks including most abundant peaks 8, 11 and 16 (~ 31–125%, *p* < 0.05) for both UVC treated samples and controls, associated likely to the normal ripening process and the additional effect of the UVC treatment while flavonol peaks 10, 12, 15 were only enhanced by UVC at day 7 (~ 67–125%, *p* < 0.05) (Fig. S1, Table 1). On the other hand, for anthocyanins on day 0 there was a decrease in peaks 1e and 2 (~ 61–63%, *p* < 0.05) and an increase in peaks 3, 4 and 5 (~ 75–458%, *p* < 0.05) for UVC treated samples compared to controls, while at day 7 there was a drastic decrease on all peaks for control samples but this decrease was to a lesser extent for UVC treated samples (*p* < 0.05) (Fig. S1, Table 1). For phenolic acids peaks 1c and 1d, changes were less evident with only coumaroyl quinic acid showing a larger increase at day 7 for UV treated fruits compared to controls (~ 52%). Accordingly, UVC led to the biosynthesis of polyphenols and induced changes in phenolic profiles in acerola.

### UVC effect on phenylalanine ammonia-lyase (PAL) and polyphenol oxidase (PPO) enzymatic activities

**Figure 4:**
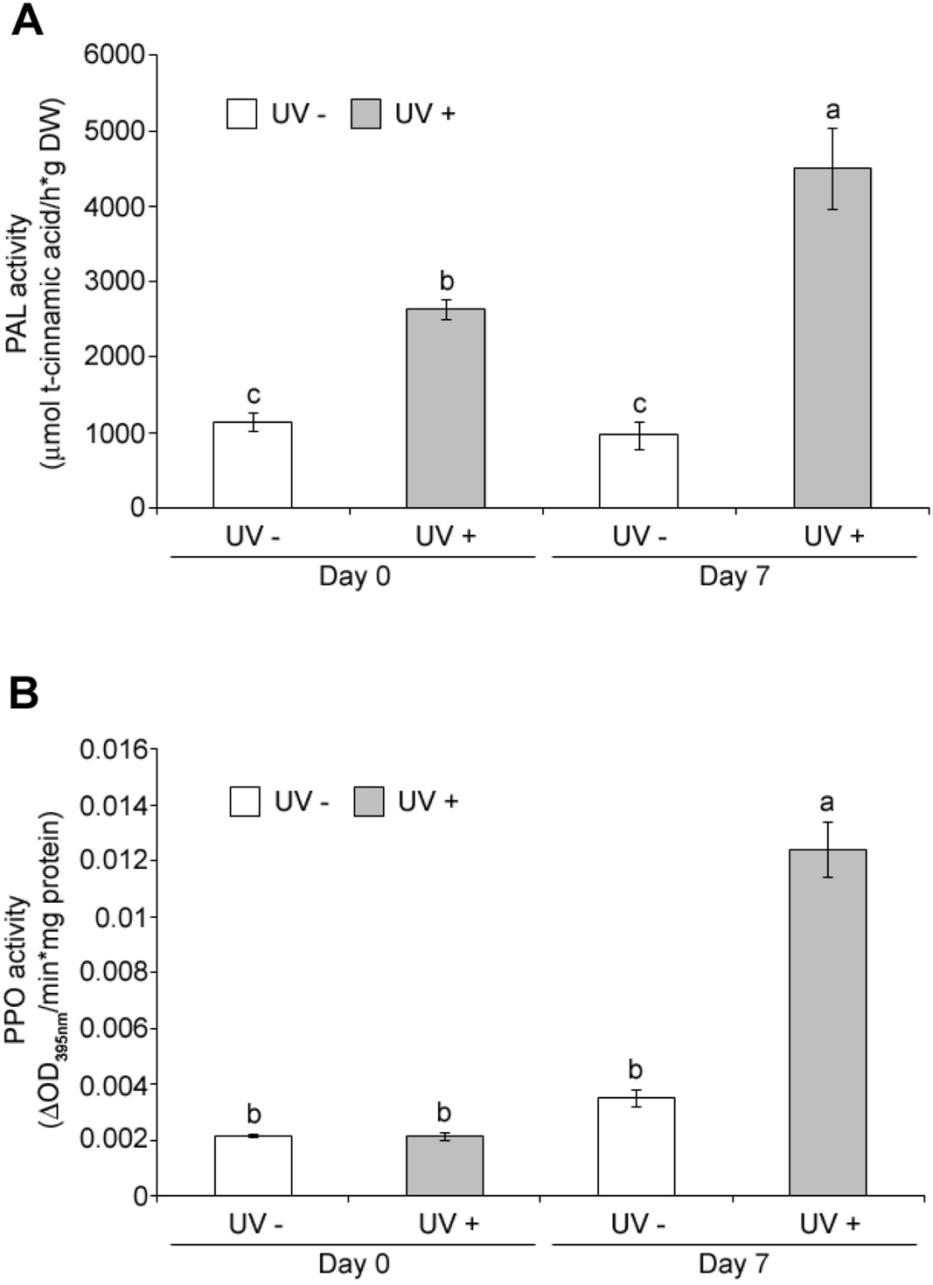
Effect of UVC on phenylalanine ammonia-lyase (PAL) and polyphenol oxidase (PPO) activity. Acerola fruits without (UV−) or with UVC treatment (UV+) were stored at 10 °C for 0 day (Day 0) or 7 days (Day 7) and subjected to the PAL assay (**A**) and the PPO assay (**B**). Data, obtained from at least three biological repeats, are shown as mean ± SE values. Different letters indicate significant differences by the ANOVA/Tukey-HSD (*p* < 0.05).

UVC induced an increase in PAL activity compared to controls at day 0 (Fig. 4a) and this increase in PAL activity due to UVC was larger at day 7 (Fig. 4a), confirming UVC induced biosynthesis of polyphenols in acerola. The effect of UVC on PPO activity was also investigated (Fig. 4b). PPO activity increased significantly only after 7 days of UVC irradiation, when compared to control. This suggests that the increase in PPO activity observed during storage of UVC treated samples enhanced phenolic conversion into *o*-quinones, which eventually will polymerize into brown pigments. However, this apparently contradicts previous results shown (Fig. 3) where UVC treated samples had significantly higher total phenolic content than control at day 7. This observed phenomenon could be explained based on PAL and PPO kinetics as described in the "Discussion" section.

### ROS production, NADPH oxidase and mitochondrial dehydrogenase activities

**Figure 5:**
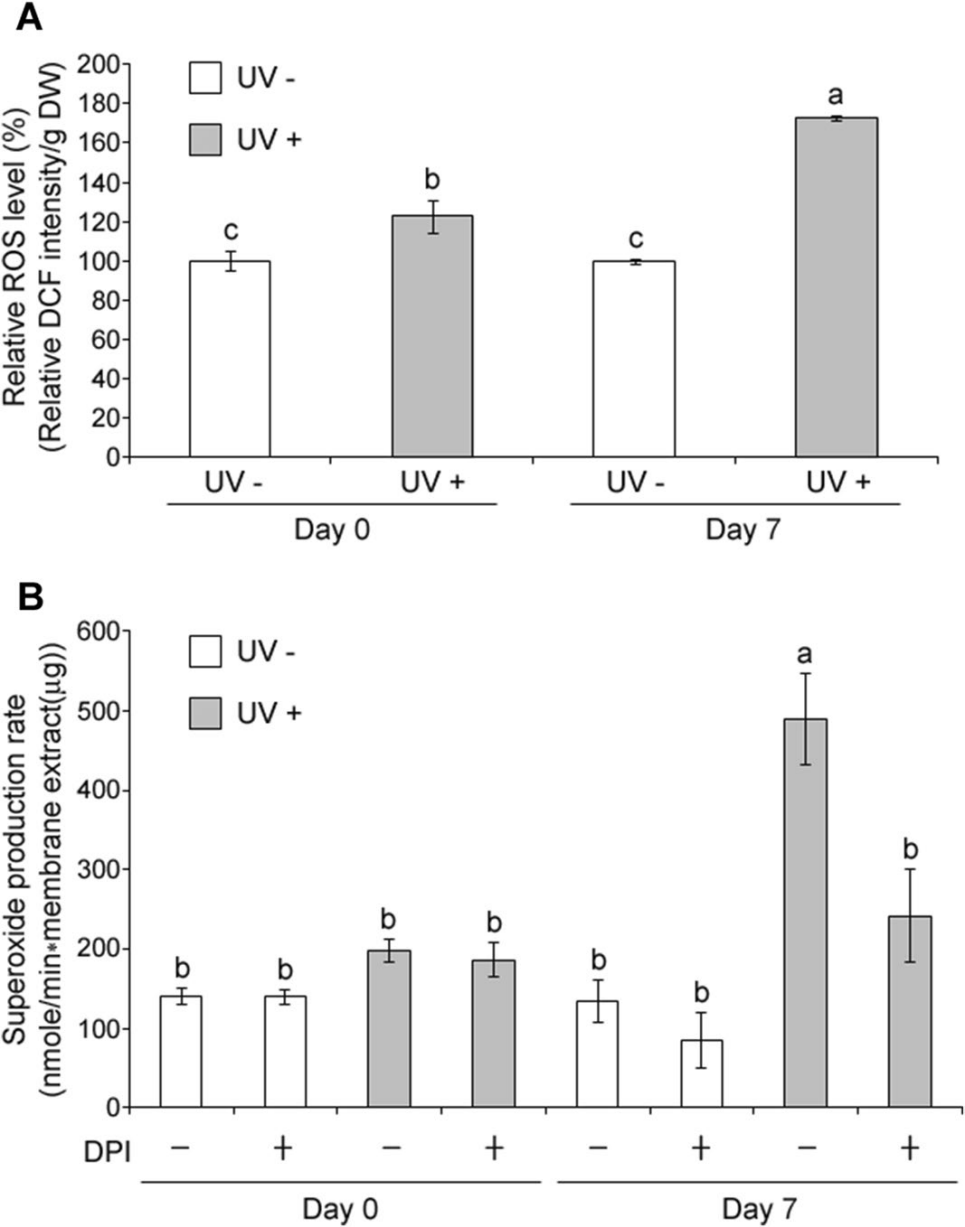
Effect of UVC on ROS production and NADPH oxidase (NOX) activity. Acerola fruits without (UV−) or with UVC treatment (UV+) were stored at 10 °C for 0 day (Day 0) or 7 days (Day 7) and subjected to ROS measurement (**A**) and NOX assay (**B**). The 350 µM DPI in (**B**) was used as a NOX inhibitor to confirm that the superoxide was generated from NOX. Data, obtained from at least three biological repeats, are shown as mean ± SE values. Different letters indicate significant differences by the ANOVA/Tukey-HSD (*p* < 0.05).

Effects of UVC on ROS level in acerola during postharvest storage were investigated. As shown in Fig. 5a, UVC significantly increased ROS levels by 30% and 70% at 0 day and 7 days, respectively, compared to controls.

**Figure 6:**
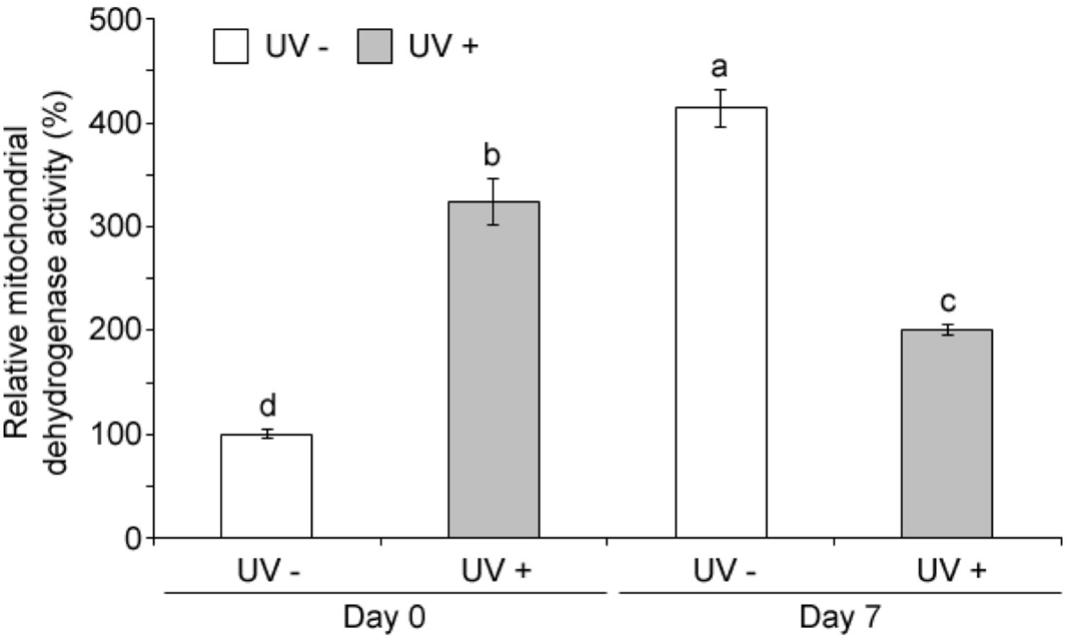
Effect of UVC on mitochondrial dehydrogenase activity. Acerola fruits, without (UV−) or with the UVC treatment (UV+) were stored at 10 °C for 0 day (Day 0) or 7 days (Day 7) and subjected to the MTS assay. Data, obtained from at least three biological repeats, are shown as mean ± SE values. Different letters indicate significant differences by the ANOVA/Tukey-HSD (*p* < 0.05).

NADPH oxidase (NOX) localized in the plasma membrane, and the respiratory electron transport chain (ETC) of mitochondria have been reported to be major sources of ROS production due to environmental stresses^33,34^. Thus, activities of NOX and of mitochondrial dehydrogenase, a component of the ETC, were evaluated. Both mitochondrial dehydrogenase and NOX activities were stimulated by UVC. Treatment increased NOX activity (Fig. 5b) only after 7 days of storage, while mitochondrial dehydrogenase increased immediately after UVC treatment, at day 0 (Fig. 6). Results suggest that ROS productions may be induced at the immediate and late times after the UVC irradiation through the mitochondrial ETC and NOX, respectively.

It is important to state that the increase observed in the MTS reduction assay which measures indirectly dehydrogenase activity is due to an increase in NADH levels that can also be associated to higher glycolysis activity besides mitochondria activity, which both together are a response associated to an increased respiratory activity.

## Discussion

### UVC alters metabolisms of phenolic compounds and vitamin C in acerola during postharvest storage

Phenolic compounds were significantly retained more in UVC treated acerola samples compared to controls after 7 days of storage (Fig. 3). In previous studies, it was proposed that the actual amounts of phenolic compounds measured result from a balance between the phenolic synthesis rate (k_s_) and their decrease or utilization rate (k_d_)^22,35^. The decrease in phenolic compounds in both UVC treated and untreated samples during 7 days of postharvest storage (Fig. 3) may be associated to a k_d_ > k_s_ and the higher content of phenolic compounds in UV treated sample after 7 days of postharvest storage compared to controls (Fig. 3) may be associated to a k_s__(UV+)_ > k_s__(UV−)_. The k_d_, associated to a decrease in phenolic compounds during postharvest storage, may be attributed partially to the phenolic oxidation by PPO^36^. In the present study, PPO activity increased significantly at 7 days after UVC irradiation (Fig. 4b), which is associated to a k_d__(UV+)_ > k_d (UV−)_ and may be caused by the induction of a plant defense system, for example, an increase of *PPO* expression responsive to stress^37^. Simultaneously, with the enhanced PPO activity due to UVC, there was an increase in PAL activity at day 0 and a significantly larger increase at day 7 confirming the biosynthesis of polyphenols in acerola with the UVC treatment (Fig. 4a). This increase in PAL activity together with PPO activity altered the profiles of phenolic acids, flavonols and anthocyanins favoring their retention in UVC treated samples compared to controls at day 7 (Fig. S1, Table 1). Thus, UVC may alter the equilibrium between the k_s_ and k_d_, resulting in the partial retention of total phenolic amount and alteration of its profile.

At day 0 there was a significant decrease in the two major anthocyanins and myricetin derivatives in UV treated acerola fruit (Table 1), likely due to the interaction of the ROS generated by UVC and these pre-existing phenolics present in the skin (e.g., anthocyanins in epidermal cells adjacent to the peel waxy cuticle). Interestingly, there was also an increase in content of some minor flavonols and anthocyanins which could be associated to the observed PAL activity increased at day 0 in UVC treated samples, (Fig. 4b, Table 1). Previous studies have reported inactive forms of PAL, which may be activated^38–41^, suggesting that in the present study UVC may revert the inactive form of PAL at day 0 and induce de novo synthesis of PAL at day 7. Further work is needed to elucidate the reverting hypothesis of inactive PAL proposed herein at day 0, and to confirm the upregulation of* PAL* and *PPO* expressions during storage. It would be important to consider other elements in further studies such as compartmentalization, isoforms, glycolization, to understand their possible role on the alteration of phenolic metabolism induced by UVC.

We identified that total vitamin C, especially AsA, was significantly retained more in UVC treated samples after 7 days of postharvest storage than in untreated ones (Fig. 1a,c,d). This also suggests that UVC may alter the equilibrium between the vitamin C synthesis rate (k_s_) and its decrease or utilization rate (k_d_), as shown in the relationship between the k_s_ and k_d_ of phenolic compounds. The observed decrease in total vitamin C and AsA in both UVC treated and control samples after 7 days (Fig. 1a,c) may be associated to a k_d_ > k_s_, but the higher retention in total vitamin C and AsA in UV treated sample after 7 days of storage compared to controls (Fig. 1a,c) would be associated to a k_s (UV+)_ > k_s (UV−)_, indicating that vitamin C biosynthesis is enhanced by UVC irradiation.

GalDH is a key enzyme producing vitamin C through the oxidation of L-galactono-1,4 lactone in the final step of the vitamin C biosynthesis^3^. In the present study, GalDH activity was enhanced immediately at day 0 after the UVC irradiation and it decreased significantly less in UV treated sample after 7 days of postharvest storage than controls (Fig. 1b) despite that GalDH activity decreased drastically for both UVC treated and untreated samples after 7 days of postharvest storage (Fig. 1b). This proves that UVC alters the equilibrium between k_s_ and k_d_ by enhancing the vitamin C biosynthesis, as k_s (UV+)_ > k_s (UV−)._

The observed enhancement of GalDH activity may be due to two possibilities: first, UVC may mediate the conformational change into a GalDH structure (i.e. phosphorylation) to have the enhanced enzymatic activity and second, may trigger the *GalDH* expression through unknown signaling pathways resulting in the increased level of GalDH proteins. However, UVC showed no significant effects on expressions of the *GMP*, *GME*, *GGP*, *GDH* and *GalDH* genes (Fig. 2). Thus, in this case GalDH activity was highly enhanced at the immediate time after UVC irradiation by UVC induced structural change rather than enzyme production.

### UVC triggers ROS productions through mitochondrial ETC and NOX for the primary and secondary signaling pathways

In this study, ROS measurement using DCFDA showed that ROS production was induced at the immediate (day 0) and late (day 7) times after the UVC irradiation (Fig. 5a).

How does ROS occur in fruits after the UVC irradiation? Here we suggest three possible answers. First, UVC may produce directly free radicals, responsible for ROS generation, through photochemical reaction because its photons have enough energy to destroy chemical bonds^42^. However, in this study, this direct effect may have little contribution to ROS production due to the short UVC irradiation pulse at a low dose (0.6 J/cm^2^).

Second, O_2_·^−^ may be produced from the mitochondria after the UVC irradiation and play an important role in a primary response to turn on secondary responses for the plant defense. This is supported by our result in that the activity of mitochondrial dehydrogenase as well as ROS production was enhanced immediately after the UVC irradiation (Figs. 5a, 6) whereas the NOX activity, one of the representative plant defense systems, was enhanced only at the late time after the irradiation treatment (Fig. 5b). The sudden ROS increase by UVC was also observed in mitochondria of *Arabidopsis* protoplasts by fluorescent microscopy^43^. The complex I of mitochondrial ETC includes dehydrogenases, whose activities can reflect the respiratory activity of mitochondria. In Fig. 6, the mitochondrial dehydrogenase activity increased significantly more in 7 days controls than in 0 day control fruits, indicating that respiratory activity increased in acerola during postharvest storage. It is known that development of deterioration and senescence of harvested commodities is generally proportional to the respiration rate and this phenomenon has been described in different crops like in potato and peach^33,44^. In contrast, the mitochondrial dehydrogenase activity was significantly lower in UVC treated acerola at 7 days after storage compared to 0 day UVC irradiated fruit (Fig. 6), which may be caused by the energy-dissipating systems as described earlier. The mitochondrial complex I also includes the GalDH responsible for vitamin C biosynthesis^45,46^, which was activated at the immediate time after the UVC irradiation (Fig. 1b), presumably through protein post translational modification. Thus, the UVC mediated excess energy in the complex I of ETC may contribute to the structural change of GalDH for the enhancement of the enzyme activity at the early time as well as the sudden ROS production, as described above.

Finally, superoxide radical is also generated by NADPH oxidase (NOX) located in the plasma membrane, as a plant response to an environmental stress such as UVC, which is proved clearly by our results that NOX activity was activated only at late time after the UVC irradiation (Fig. 5b), resulting in increased ROS production at that late time (Fig. 5a). Furthermore, it is suggested that the enhanced NOX activity at late time after the UVC irradiation may be attributed to an increased Ca^2+^-mediated signaling^47^ and possibly *NOX* gene expression^48^. The fate of the O_2_·^−^, triggered at late time, would be as follow: the NOX-generated O_2_·^−^ is converted to H_2_O_2_ by SOD, which may function as a key ROS mediating signal pathway to induce expression of genes such as *PAL* and *CHS* responsible for biosynthesis of secondary metabolites with antioxidant capacity^49–51^.

**Figure 7:**
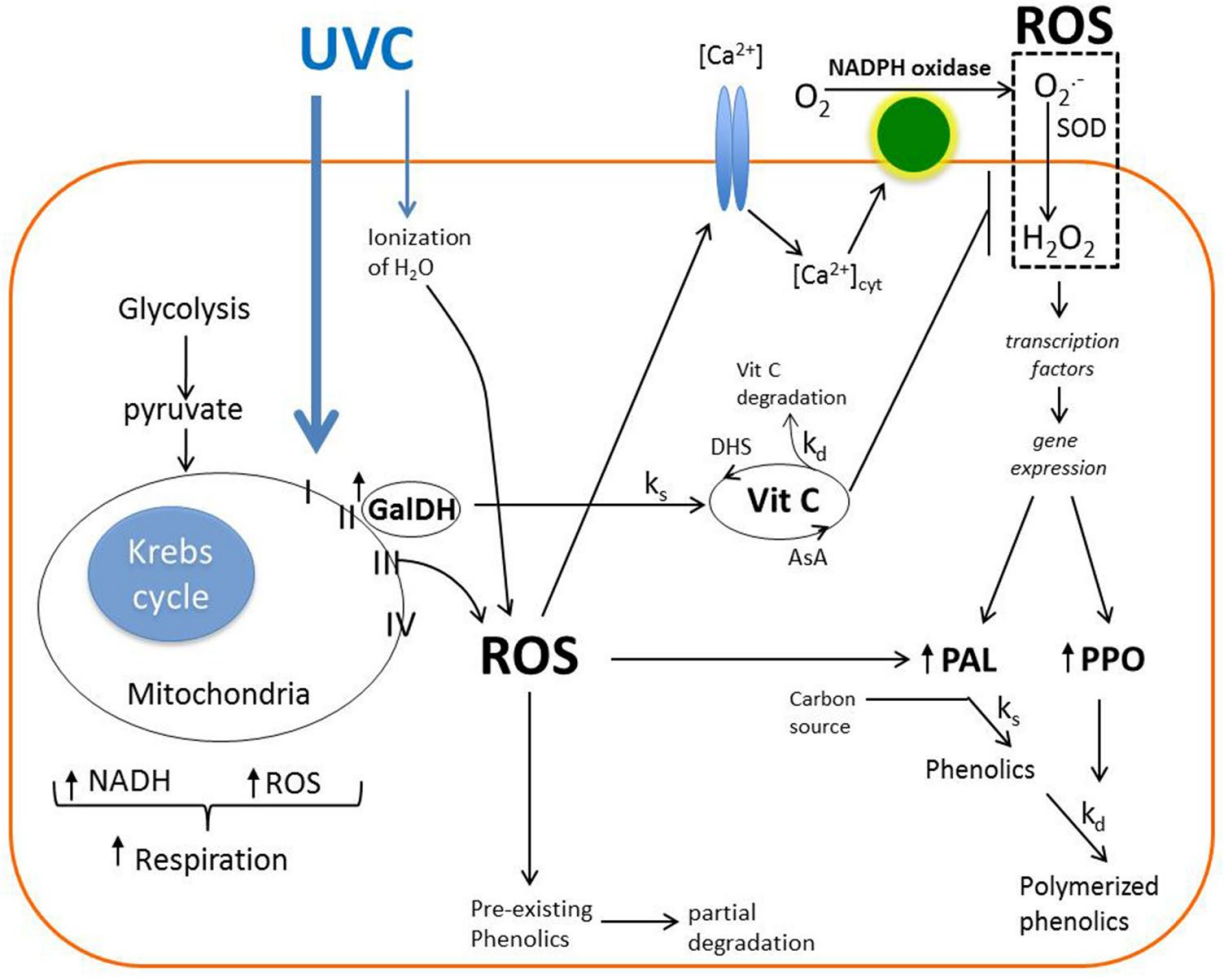
Mechanistic model of UVC stress signaling pathways in acerola. At the immediate UVC response, an initial burst of ROS occurs mediated mainly by a mitochondrial energy dissipating system and secondly a partial ionization of water. The latter mechanism may contribute to the overall pool of ROS generated, while the former mechanism generates an increase in the glycolysis and mitochondria activity (dehydrogenase activity) generating high levels of NADH and ROS. Simultaneously, GalDH is activated promoting the synthesis of ascorbic acid (k_s_). The generated mitochondrial ROS triggers an early response of increased levels of [Ca^2+^]_cyt_ which activates NADPH oxidase generating a second burst of ROS at late time. This in turn triggers an increase in gene and protein expression and activity of PAL and PPO. The final phenolics content under UVC at late time is dependent on the relative kinetics of phenolic synthesis (k_s_) and decrease (k_d_) associated to PAL and PPO. The immediate increased levels of mitochondrial ROS also induce a partial degradation of pre-existing phenolics and an early activation of inactive PAL while the generated ascorbic acid play a role of modulating ROS levels from NADPH oxidase sources at late time causing vitamin C degradation (k_d_) and contributing ultimately to the overall amount of phenolics synthesized in the late response. Reactive oxygen species (ROS); superoxide dismutase (SOD); phenylalanine-ammonia-lyase (PAL); polyphenol oxidase (PPO); L-galactono-1,4-lactone dehydrogenase (GalDH); k_s_ (rate of synthesis); k_d_ (rate of decrease or utilization).

In conclusion, results showed that hormetic doses of UVC irradiation helps in the retention of vitamin C and phenolic content in acerola by altering vitamin C and phenolic metabolism through an increase in mitochondrial activity and a ROS-mediated mode of action. Furthermore, results also supplied new insights into understanding the mechanism by which UVC irradiation enhances the antioxidant system in fruits, as described in the proposed model in Fig. 7. In this model UVC induces excess energy in the mitochondrial ETC, leading to a ROS increase and enhanced GalDH activity at the immediate time after treatment. The early ROS functions as a primary signal molecule (ROS initial burst) to turn on plant responses like plant defense systems including induction of NOX and PAL activity, and the early activation of GalDH may increase vitamin C biosynthesis, preventing decrements in its concentration by altering the equilibrium between the k_s_ and k_d_ of vitamin C metabolism in acerola during postharvest storage. Furthermore, the NOX-generating ROS at late time after UVC irradiation (ROS second burst) may play an important role as a secondary signaling molecule to induce expression of genes such as *PAL* and *PPO* responsible for the biosynthesis of phenolic compounds and their utilization^52^, altering the equilibrium between the k_s_ and k_d_ of the phenolic metabolism in acerola during postharvest storage. Altogether, our study may contribute to the development of advanced postharvest techniques based on hormetic dose of UVC to prolong postharvest life of commercial fruits.

## Materials and methods

### Plant material

Acerola fruits (*Malpighia emarginata* DC) were obtained in 2013 from a local market (Fortaleza, Ceara State, Brazil) where all fruits are sold one day after harvest at physiological maturity (when the fruit color is half green and half orange-red). Before UVC treatments, fruits were sorted by color and size, and then washed with distillated water, dried and divided into two groups including controls and UVC treatment, respectively. Fruits selected showed moisture content of ~ 90%, ~ 10 soluble solids, and color average values of Hue = 25°, L = 70 and Chroma = 25.

### UVC treatment and storage

The UVC treatment was done at EMBRAPA Agroindústria Tropical (Fortaleza, Brazil) using a XeMaticA–2LXL System (SteriBeam Systems GmbH, Germany). Fruit were treated twice with UVC pulses of 0.3 J cm^−2^ (total dose of 0.6 J cm^−2^). After the treatment, UVC treated and non-treated control fruits were immediately processed, consisting of samples at day 0, and the other half amount of samples were stored in a refrigerator at 10 °C for 7 days, when they were processed. The processing consisted of homogenization using a blender followed by freeze-drying at − 55 °C in a L202 freeze dryer (Liotop, São Carlos, Brazil). Freeze-dried samples were grounded in liquid N_2_ to obtain fine powders, which were used for all analyses performed at Texas A&M University. The selected UV dose and storage dates were defined in preliminary studies based on large differences in ROS, enzyme activities, vitamin C and polyphenol synthesis.

### Quantification of total vitamin C (ascorbic acid), AsA and DHA

The total vitamin C, reduced ascorbic acid (AsA) and dehydroascorbic acid (DHA) measurements were adapted from the method described previously by Gillespie and Ainsworth^53^. A 50 mg of sample powder was homogenized with 2 ml of 5% (w/v) trichloroacetic acid (TCA), filtered through four layers cheese cloths and then centrifuged at 16,000×*g* for 10 min at 4 °C. The supernatant was used for the total vitamin C and AsA measurements.

For the AsA quantification, a reaction mixture containing 50 µL supernatant, 25 µL 100 mM phosphate buffer (pH 7.7), 50 µL 10% (w/v) TCA, 50 µL 44% (w/v) phosphoric acid, 50 µL 4% (w/v) 2,2′-bipyridyl and 25 µL 3% (w/v) FeCl_3_ were incubated at 37 °C for 60 min and then absorbance was read at 525 nm. The total vitamin C was measured by incubating a reaction mixture containing 50 µL supernatant, 12.5 µL 100 mM phosphate buffer (pH 7.7) and 12.5 µL 0.2 mM DTT, at room temperature for 10 min. After incubation, 50 µL 10% (w/v) TCA, 50 µL 44% (w/v) phosphoric acid, 50 µL 4% (w/v) 2,2′-bipyridyl and 25 µL 3% (w/v) FeCl_3_ were added to the final reaction mixture, which was incubated at 37 °C for 60 min. Absorbance was monitored at 525 nm. DHA was quantified from the difference between AsA and total vitamin C. Ascorbate was used to obtain a standard curve and the results were expressed as mg total vitamin C⋅100 g^−1^ DW, mg AsA⋅100 g^−1^ DW and mg DHA⋅100 g^−1^ DW.

### L-galactono-1,4-lactone dehydrogenase (GalDH) assay

GalDH activity was determined as reported by Ôba et al*.*^54^. First, as it is located in the mitochondrial membrane, the mitochondrial fractions were prepared by homogenizing 300 mg of sample powder in 4 mL of ice cold extraction solution, containing 0.1 M Tris–HCl (pH 7.4), 0.4 M sucrose, 2% (w/v) PVP and 50 mM β-mercaptoethanol, for 2 min. The final solution was filtered through four layer of cheese-cloth and then centrifuged at 500 g for 10 min at 4 °C. The collected supernatant was centrifuged again at 10,000×*g* for 10 min at 4 °C to precipitate mitochondrial pellets, which were resuspended in a wash solution containing 0.1 M Tris–HCl buffer (pH 7.4) and 0.4 M sucrose and then centrifuged at 10,000×*g* for 10 min at 4 °C. This washing step was performed twice. The final pellet was used as a mitochondrial fraction. For the GalDH enzymatic assay, a protein extract was prepared from the mitochondrial fraction, which was resuspended in 0.1 M Tris–HCl buffer (pH 7.4) containing 0.4 M sucrose, 1 mM EDTA and 0.1% (v/v) Triton X-100, incubated for 1 h at 4 °C and then centrifuged at 10,000×*g* for 10 min at 4 °C. The supernatant was used as mitochondrial proteins extract for the GalDH enzymatic assay. The reaction mixture was initially composed of 10 µL protein extract and 80 mM cytochrome c. After its incubation at room temperature for 10 min, the substrate 4.6 mM l-galactono-1,4-lactono was added to start reaction. The increase of absorbance at 550 nm, caused by the Cyt *c* (ε = 21.6 mM^−1^ cm^−1^) reduction, was monitored every 1 min for 30 min. One unit of activity is defined as the amount of enzyme required for oxidizing 1 nmol of l-galactono-1,4-lactono (equivalent to the formation of 2 nmol of reduced Cyt *c*) per min.

### Total RNA preparation and real-time qRT-PCR analysis

After grinding the acerola samples under liquid nitrogen, 100 mg of fine powders were subjected to the total RNA preparation, which was performed using the RNeasy Plant Mini Kit (Qiagen, Mississauga, ON) according to the manufacturer's instructions. The total RNAs were treated with DNase I to avoid DNA contamination and then the RNA quality and quantity were examined by both of agarose gel electrophoresis and a NanoDrop ND-1000 spectrophotometer (NanoDrop Technologies, Willmington, DE). For cDNA synthesis, 200 ng RNAs were reverse-transcribed into cDNA using the SuperScript III first-strand synthesis supermix (Invitrogen, Carlsbad, CA), following the manufacturers protocol. cDNAs were used for the real-time qRT-PCR analyses, which were performed using Power SYBR Green PCR Master Mix (Applied Biosystems, Foster City, CA), following the manufacturer’s instructions. cDNA amplification was carried out using a 7900 HT Sequence Detection System (Applied Biosystems, Foster City, CA). The primer sets are listed in Table S2 and were provided by Integrated DNA Technologies (IDT, Coralville, IA). The relative expression of each gene was normalized by *Actin* (forward primer 5′-GGGCATCGGAAACGTTCAGCACCG-3′, reverse primer 5′-CGTTCACCACTACTGCTGAACGAG-3′);^55^ and was calculated following the comparative Ct method (ΔΔCt), also known as the 2^− ΔΔCt^ method^56^.

### Phenolics profiling by LC–MS and quantification of individual and total phenolics

To obtain the phenolic profile of the samples, 1.5 g of the freeze-dried sample powder was homogenized overnight at 4 °C with 50 mL of 85% methanol to obtain the crude extract. This extract was fractionated into phenolic acids (F1), anthocyanins (F2), flavonols (F3), and procyanidins/polymeric anthocyanins (F4) by solid-phase extraction using C18 cartridges as reported previously^57^. Initially, the methanolic extract was evaporated and the recovered aqueous extract was adjusted to pH 7.0 with 5 N NaOH. A total of 10 mL of extract was loaded in SEP Pack C18 cartridge (55–105 μm, Waters Corp., Milford, MA) previously conditioned to pH 7.0 with 25 mL 100% methanol and 25 mL nanopure water (pH 7.0). The neutral phenolics were absorbed to cartridge, while the phenolic acids were not. The cartridge was then washed with 25 mL of water (pH 7.0), which was combined with the non-absorbed phenolics fractions and had its pH adjusted to pH 2.0. This mixture of compounds was loaded into a second cartridge previously conditioned to pH 2.0 with 25 mL of 100% methanol and 25 mL of nanopure water at pH 2.0. Phenolic acids bound to the matrix of the second cartridge (F1) were later eluted with 25 mL of 100% methanol. After the pH was adjusted to 2.0 in the first cartridge, elution of anthocyanins was accomplished by passing 25 mL of 16% acetonitrile at pH 2.0 (F2). The flavonols were eluted using 25 mL of 100% ethyl acetate (F3) and the anthocyanin polymers using 25 mL of 100% methanol (F4). Fractions were completely evaporated at 45 ºC using a speed vac (Savant SC100, MN, USA) and recovered in 85% methanol at final concentration of 5 mg/mL for the LC–MS work. Fraction yields were F1—9.9 mg; F2—18.3 mg; F3—105.5 mg and F4—199.9 mg.

Individual compounds were identified on the basis of retention time, UV spectra, and their mass-to-charge ratio using LC–MS/MS by injecting 10 µL of sample. Chromatographic separations were performed on a LCQ Deca XP Max MS^n^ system (Thermo Finnigan, San Jose, CA, USA) equipped with an autosampler, a Surveyor 2000 quaternary pump and a Surveyor UV 2000 PDA detector using a Hydro-RP18 Phase (150 mm × 4.6 mm × 3 mm, Phenomenex, Torrance, CA, USA, particle Sizes (4 µm) and pore size of 100 Å) and a guard column of the same chemistry. Elution gradients were performed with solvent A, which consisted of acetonitrile and methanol (1:1 containing 0.5% formic acid) and solvent B (water containing 0.5% formic acid). The applied elution conditions were: 0–2 min, 2% A, 98% B; 3–5 min, 5%A, 95% B, 5–30 min, 20% A, 80% B; 30-72 min, 35% A, 65% B; 72–83 min, 100% A, 0% B; 83–85 min was held isocratic, 100% A; 87-90 min 2% A, 98% to the starting condition. The chromatograms were monitored at 520, 330, 280 and 210 nm, and complete spectral data were recorded in the range 200–600 nm. Nitrogen was used as desolvation gas at 275 °C and a flow rate of 60 L/h, and He gas was used as damping gas. Mass spectra were obtained on a MS Finnigan LCQ Deca XP Max, Ion trap mass spectrometer coupled at the exit of the diode array detector and equipped with a Z-spray ESI source, and run by Xcalibur version 1.3 software (Thermofinnigan-Surveyor, San José, USA). A potential of 6.8 V was used on the capillary for positive ion mode. Spray voltage of 4.57 kV and the source block temperature was held at 255 °C.

Individual phenolics were quantified from chromatogram areas (Table S1) using standard curves prepared in the range of 0.1–100 ug/mL of gallic acid (for gallic acid), chlorogenic acid (for coumaroyl quinic acid), cyanidin (for anthocyanins), kaempferol (for kaempferol derivatives), quercetin (for isorhamnetin and quercetin derivatives) and myricetin (for myricetin derivatives). Individual phenolic content was expressed as mg of the corresponding phenolic equivalent/100 g DW. Total phenolic values were determined by the sum of the concentration of individual phenolics and expressed as mg phenolics/100 g DW.

### Reactive oxygen species (ROS) measurement

ROS were measured using 2′,7′-Dichlorofluorescin diacetate (DCFDA) method as described previously^58^. A 100 mg of fine powder from samples were homogenized with 700 µL of 10 mM Tris–HCl (pH 7.2) and centrifuged at 12,000× g for 20 min at 4 °C. After centrifugation, the supernatant was used for ROS measurement; the mixture containing 10 × diluted sample and 10 µM DCFDA was incubated in the dark for 10 min and fluorescence was immediately read at 485 nm for excitation and 528 nm for emission on a 96-well microplate reader (Synergy HT, Bio-Tek Instruments, Inc., Winooski, VT). The result was represented as a relative fluorescent intensity per gram (g^−1^ DW) between control and treatment samples.

### NADPH oxidase (NOX) assay

The membrane protein preparation and NOX activity assay were performed as described in a previous report by Agarwal et al*.*^59^. For membrane protein preparation, 500 mg of fine powder were homogenized with the extraction buffer containing 50 mM Tris–HCl (pH 7.2), 0.25 M sucrose, 3 mM EDTA, 1 mM DTT, 3.6 mM L-cysteine, 0.1 mM MgCl_2_, 0.6% PVP and 0.5 mM PMSF, filtered through four layers of cheese cloths and then, centrifuged at 10,000×*g* for 45 min at 4 °C. For precipitation of membrane proteins, the supernatants were centrifuged at 65,000×*g* for 30 min at 4 °C and then pellets including the membrane proteins were recovered in the suspension buffer including 10 mM Tris–HCl (pH 7.2), 0.25 M sucrose and 0.5 mM PMSF. Protein amount from suspension was quantified and used for the NOX assay.

NOX assay was performed with 50 mM Tris–HCl buffer (pH 7.2), 100 µM NADPH, 0.5 mM cytochrome c and 10 µL membrane proteins with or without 350 µM DPI. The superoxide generation rate (nmole/µg of extract per min) was calculated by the superoxide-induced reduction rate of ferricytochrome c to ferrocytochrome c (extinction coefficient = 21.6 mM^−1^ cm^−1^), monitored spectrophotometrically at 550 nm every 2 min for 1 h. The reaction without the membrane proteins or NADPH was used as negative controls. The results were represented as cytochrome c (cyt c) reduction rate min^−1^ µg protein^−1^.

### Phenylalanine ammonia-lyase (PAL) assay

PAL assay was carried out measuring *trans*-cinammic acid by LC–MS based on the protocol by Kim et al.^60^ with some modifications. To 0.4 g of freeze-dried acerola sample, 0.4 g of polyvinylpyrrolidone (PVP) was added followed by 16 mL of 50 mM borate buffer pH 8.5, containing 5 mM β-mercaptoethanol. The mixture was homogenized for 30 s and filtered through 4 layers of cheesecloth followed by centrifugation at 12 000 × g at 4 °C for 20 min and then 500 µL of the supernatant were filtered through a Microcon centrifugal filter (Millipore, 10,000 MWCO, Germany). This filtrate was used for PAL activity determinations. For each treatment, the reaction mixture consisted of 235 µL buffer, 80 µL raw extract of acerola filtrate and 35 µL of 100 mM L-phenylalanine (L-phe), while for the controls water was used instead of L-phe. The reaction was incubated at 37 °C for 60 min and stopped by adding 35 µL of glacial acetic acid. Reaction product (*trans*-cinammic acid) was extracted by adding and mixing thoroughly 750 µL of ethyl acetate. Thereafter, 500 µL was removed and dried in a speedvac (Labconco, Kansas City, MO), and re-dissolved in 50 µL of 50% methanol and analyzed for *trans*-cinnamic acid quantification by LC–MS.

*Trans*-cinnamic acid was determined by injecting the above methanol extract (10 µL) in the HPLC/MS system. Compounds were separated on a 4µ Hydro-RP-80A° 150 mm × 2 mm, on a reverse phase column (Luna, Phenomenex, Torrance, CA, USA) with a guard column and maintained at 25 °C. The mobile phases consisted of water (phase A) and methanol:acetonitrile (60:40, v:v, phase B) both adjusted to pH 4.5 with formic acid. The gradient solvent system was 0/100, 3/90, 10–15/75, 16/70, 25/25, 30/10, 32–34/0, 35–40/100 (min/% phase A) at a constant flow rate of 0.2 mL/min. Mass spectra were obtained on a Thermo-Finnigan LCQ Deca XP Max Ion Trap Mass Spectrometer coupled at the exit of the PDA and equipped with a *Z*-spray ESI source, and run by Xcalibur version 1.3 software (ThermoFinnigan Surveyor, San José, CA, USA). Separations were conducted using the Phenomenex (Torrance, CA, USA) Synergi 4 μm Hydro-RP 80A (2 mm × 150 mm) with a C_18_ ward column, and a flow of 200 µL/min from the PDA eluent was directed to the ESI interface using a flow-splitter. The chromatograms were monitored at 330, 280, and 210 nm; and complete spectral data were recorded in the range 200–600 nm. Nitrogen was used as desolvation gas, at 275^◦^C and a flow rate of 60 L/h, and He gas was used as damping gas. A potential of 6.8 V and a spray voltage of 4.5 kV was used on the capillary for positive mode. A standard curve was constructed with *trans*-cinnamic acid and results were reported as µmol *t*-cinnamic acid/h g DW.

### Polyphenol oxidase (PPO) assay

Extracts preparation assay of PPO were performed as described by Sojo et al*.*^61^. 100 mg of the fine powders were homogenized in 2 mL of 0.1 M potassium phosphate buffer (pH 7.0) containing 2% PVPP and 4% Triton X-100 with Ultraturrax (IKA, Staufen, Germany), followed by 30 min of incubation at 4 °C. The final solution was centrifuged at 10,000×*g* for 15 min at 4 °C and the supernatant was crude extract for enzymatic assay.

The activity was measured by monitoring the phenolic degradation at 395 nm, as described by Wissemann & Lee^62^. The reaction mixture consisted of the extract prepared above, 0.1 M potassium phosphate buffer (pH 7.0) and 0.03 M catechin. A unit of enzymatic activity was represented as ΔO.D min^−1^ mg of protein^−1^.

### Mitochondrial dehydrogenase activity measurement by MTS assay

For the measurement, MTS tetrazolium assay was performed using the Cell Titer 96® AQueous Non-Radioactive Cell Proliferation Assay kit (Promega) according to the manufacturer’s instruction. The fraction including native mitochondria was prepared as described in the measurement of GalDH activity above and was resuspended in a buffer, which consisted of 50 mM Tris–HCl (pH 7.4) and 0.4 M sucrose. 30 µL of the mitochondrial fraction were incubated with 20 mM potassium phosphate (pH 7.4) buffer including 20 mM succinate and 50 µL MTS solution at 37 °C for 2 h and then the production of formazan was measured by absorbance at 490 nm, which was directly proportional to the mitochondrial dehydrogenase activity. Protein content was quantified and used to normalize amount of mitochondria extracted from control and treated samples. Results were expressed as relative mitochondrial dehydrogenase activity (%) per protein amount (µg).

### Protein quantification

The total protein was quantified by the colorimetric method with bicinchoninic acid solution using the Pierce® BCA Protein Assay Kit (Pierce, Rockford, IL) according to the manufacturer’s instruction, with absorbance monitored at 562 nm.

### Statistical analyses

Three biological replicates were performed. Each biological replicate was composed of 20 fruits, giving a total of 60 fruits per each treatment. The data were analyzed using student’s t-test or one-way analysis of variance (ANOVA) followed by Tukey-HSD test, using the software JMP pro v10.0. Results were expressed as means ± standard errors (SE). Different letters show significant differences (*p* < 0.05).

## Supplementary information

Supplementary Information.
