# Supplementary material for: UVC light modulates vitamin C and phenolic biosynthesis in acerola fruit: role of increased mitochondria activity and ROS production
Source: Sci Rep. 2020 Dec 15;10:21972. doi: 10.1038/s41598-020-78948-1 (PMC7738507; doi:10.1038/s41598-020-78948-1)
Supplement: Supplementary file 1 — Supplementary Information. [file 41598_2020_78948_MOESM1_ESM.docx]

***Supplementary Information***

**Ultraviolet-C light modulates vitamin C and phenolic biosynthesis in acerola fruit: Role of increased mitochondria activity and ROS production**

Marcela Cristina Rabelo^1,2#^, Woo Young Bang^1,3#^, Vimal Nair^1#^, Ricardo Elesbao Alves^1,4^, Daniel A. Jacobo-Velázquez^5^, Shareena Sreedharan^1^, Maria Raquel Alcântara de Miranda^2^ and Luis Cisneros-Zevallos^1*^

^1^Department of Horticultural Sciences, Texas A&M University, College Station, TX 77845, USA

^2^Department of Biochemistry and Molecular Biology, Universidade Federal do Ceará, Fortaleza, CE, Brazil

^3^National Institute of Biological Resources (NIBR), Environmental Research Complex, Incheon 404-708, Republic of Korea

^4^Embrapa Tropical Agroindustry, Fortaleza, CE, Brazil

^5^Tecnológico de Monterrey, Escuela de Ingeniería y Ciencias, Centro de Biotecnología FEMSA, Eugenio Garza Sada 2501 Sur, CP 64849 Monterrey, NL, México

^#^These authors, WYB, VN and MCR, contributed equally to this work

*Corresponding author:

Luis Cisneros-Zevallos

lcisnero@tamu.edu

Phone: 979-8453244

Fax: 979-8450627

**Figure S1. Liquid chromatograms of F3 and F4 fractions extracted from acerola fruits.** Crude extracts were prepared from acerola fruits without (UV-) or with UVC treatment (UV+) that were stored at 10 °C for 0 day (Day 0) or 7 days (Day 7). Extracts were fractionated into phenolic acids (F1), anthocyanins (F2), flavonols (F3), and procyanidins/polymeric anthocyanins (F4) by solid-phase extraction using C18 cartridges as previously described (*1*). Subsequently, each fraction was subjected to liquid chromatography for separation of compounds. No peaks associated to phenolics were found in F1 and F2 fractions while F3 at 330 nm (A) and F4 at 520 nm (B) showed several peaks, identified by mass spectrometry as shown in Table S1 and the supplementary information.

**A)**

**UV- (Day0, Fraction F3)**


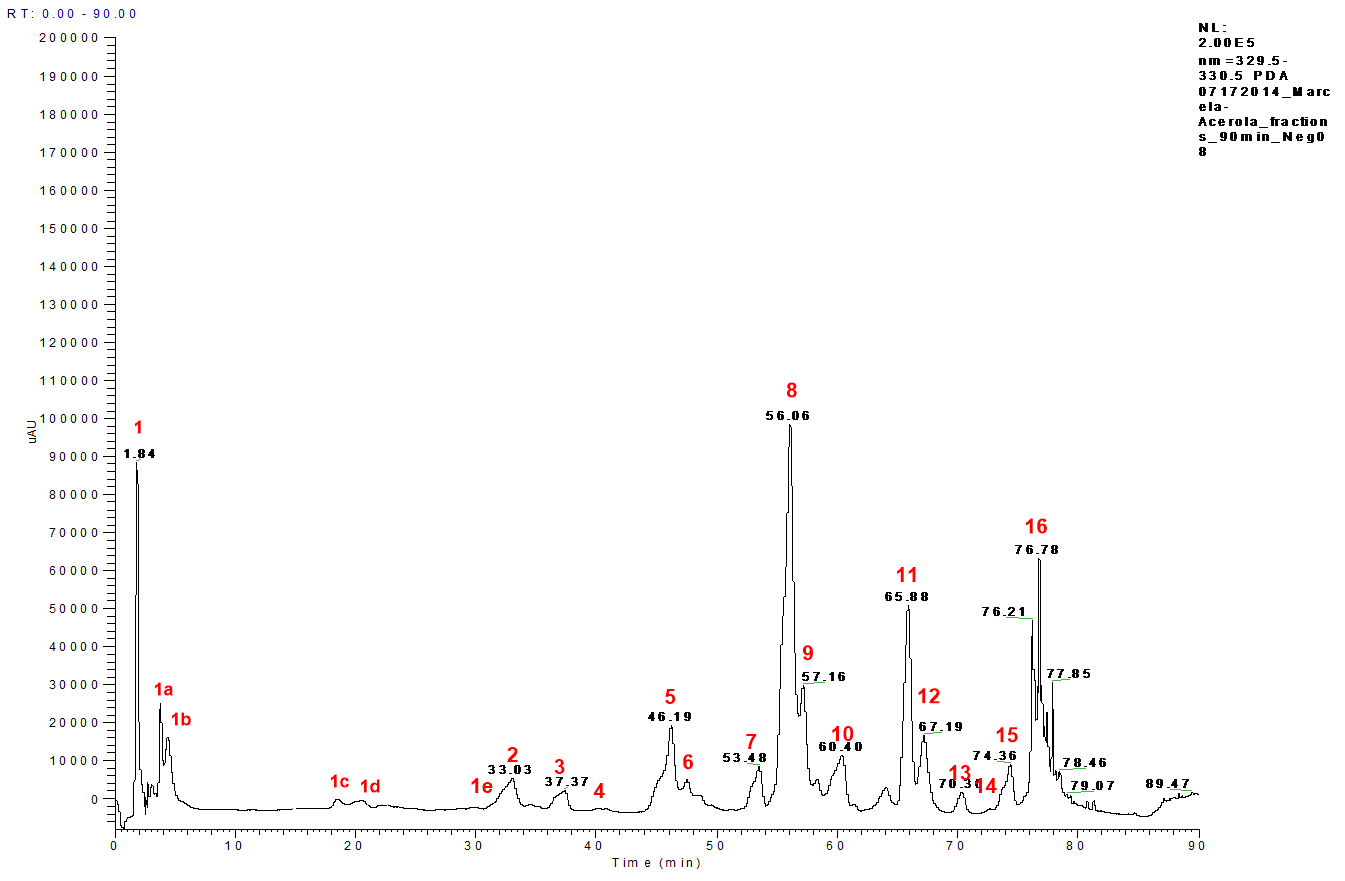


**UV+ (Day0, Fraction F3)**


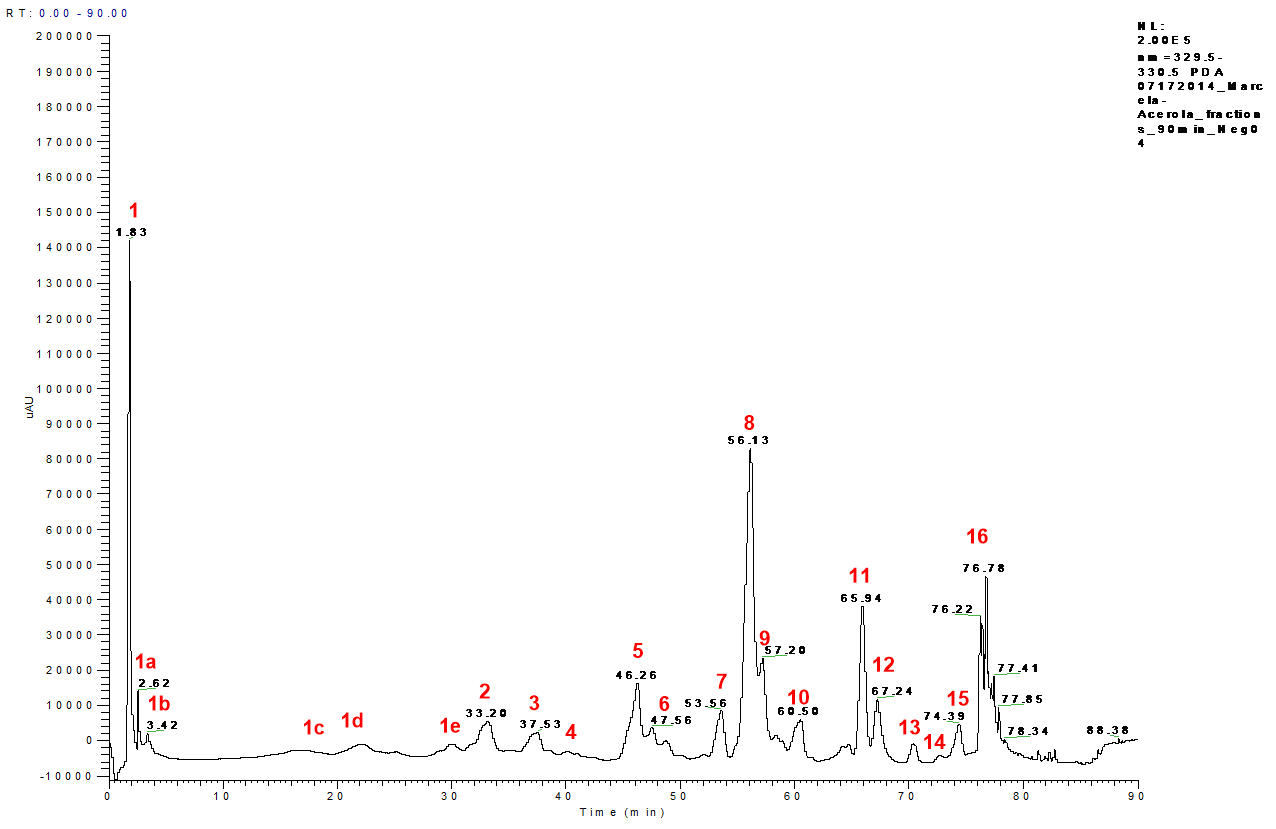


**UV- (Day7, Fraction F3)**


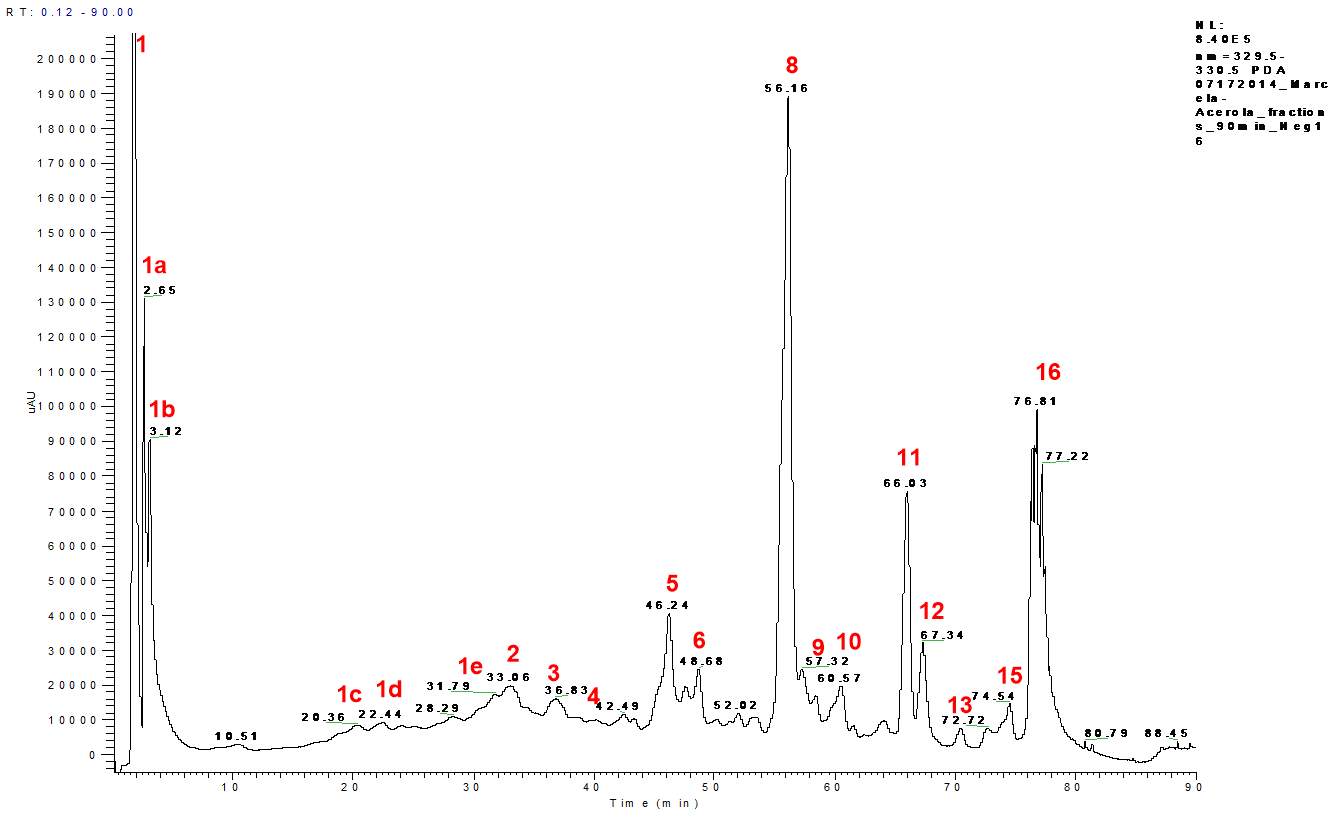


**UV+(Day7, Fraction F3)**


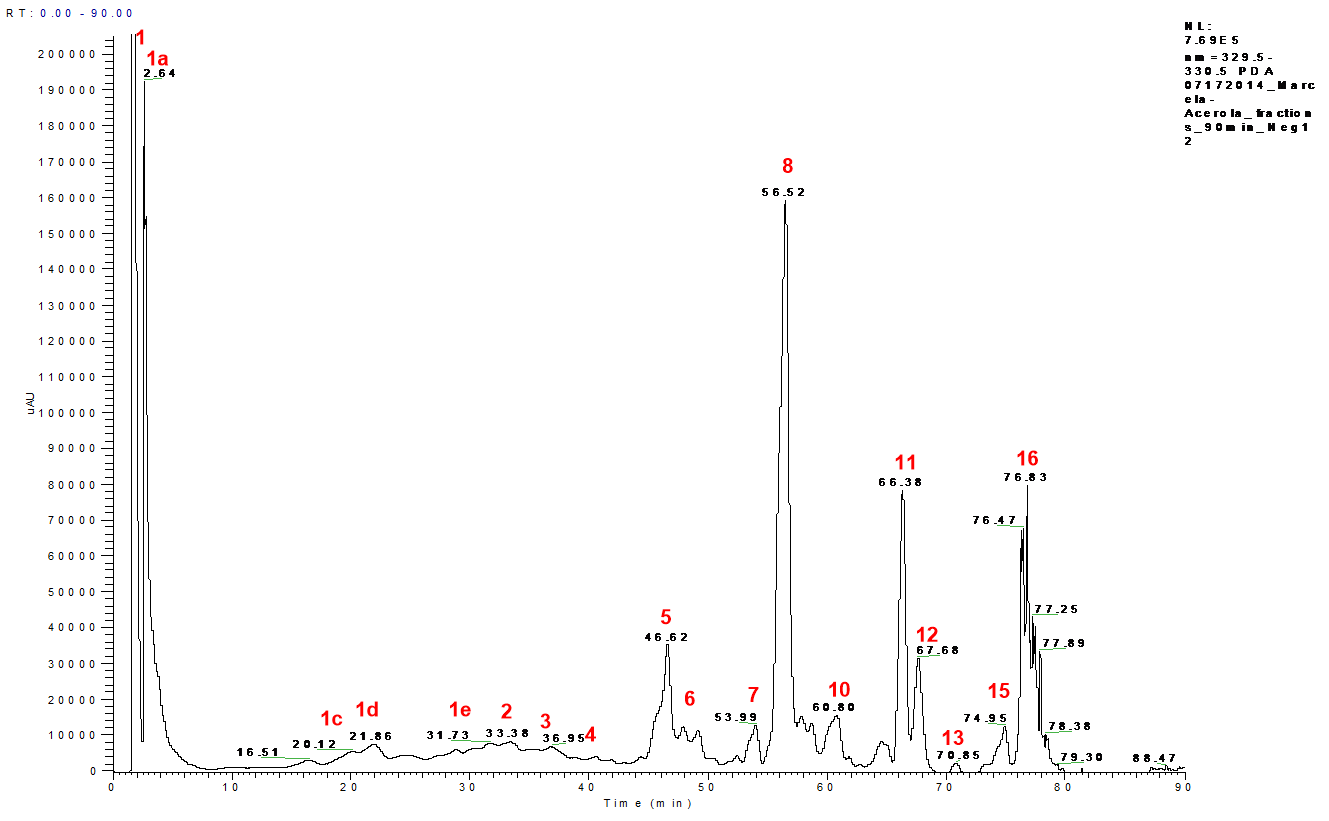


**B)**

**UV- (Day0, Fraction F4)**


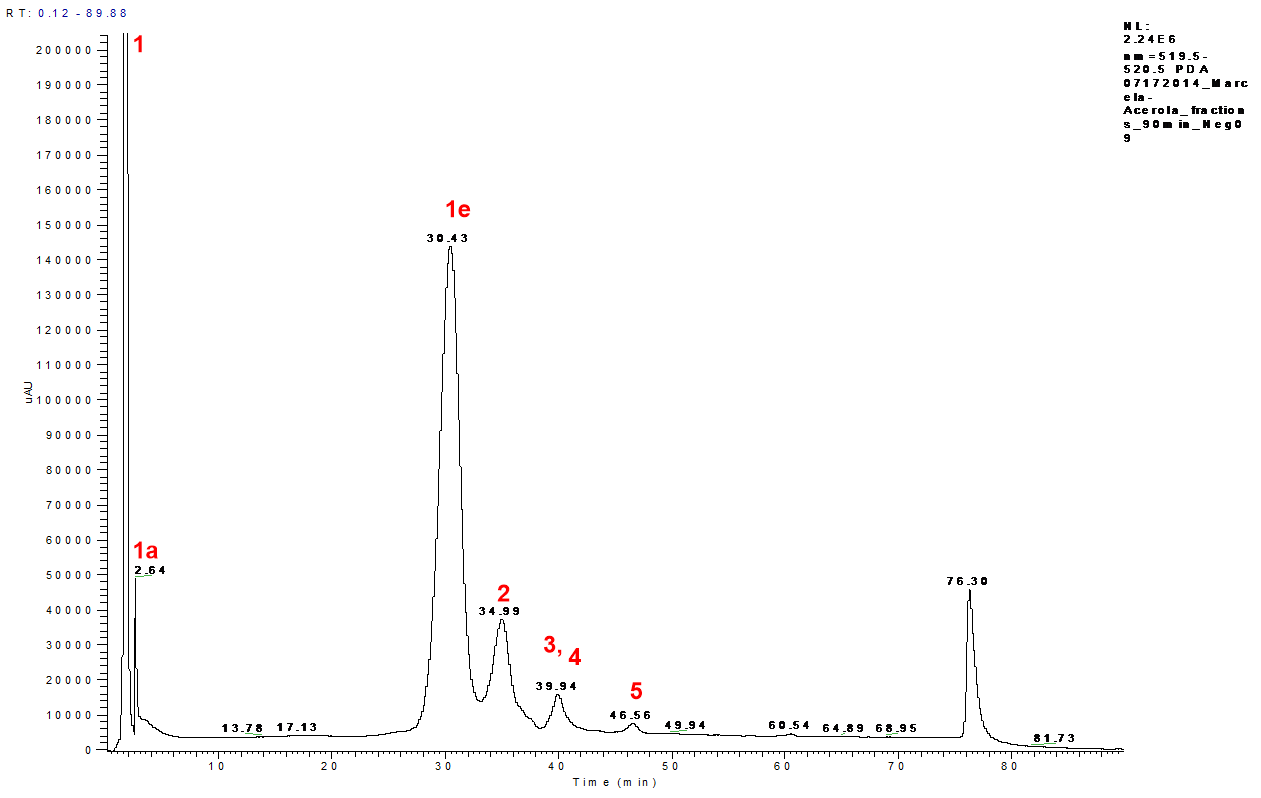


**UV+ (Day0, Fraction F4)**


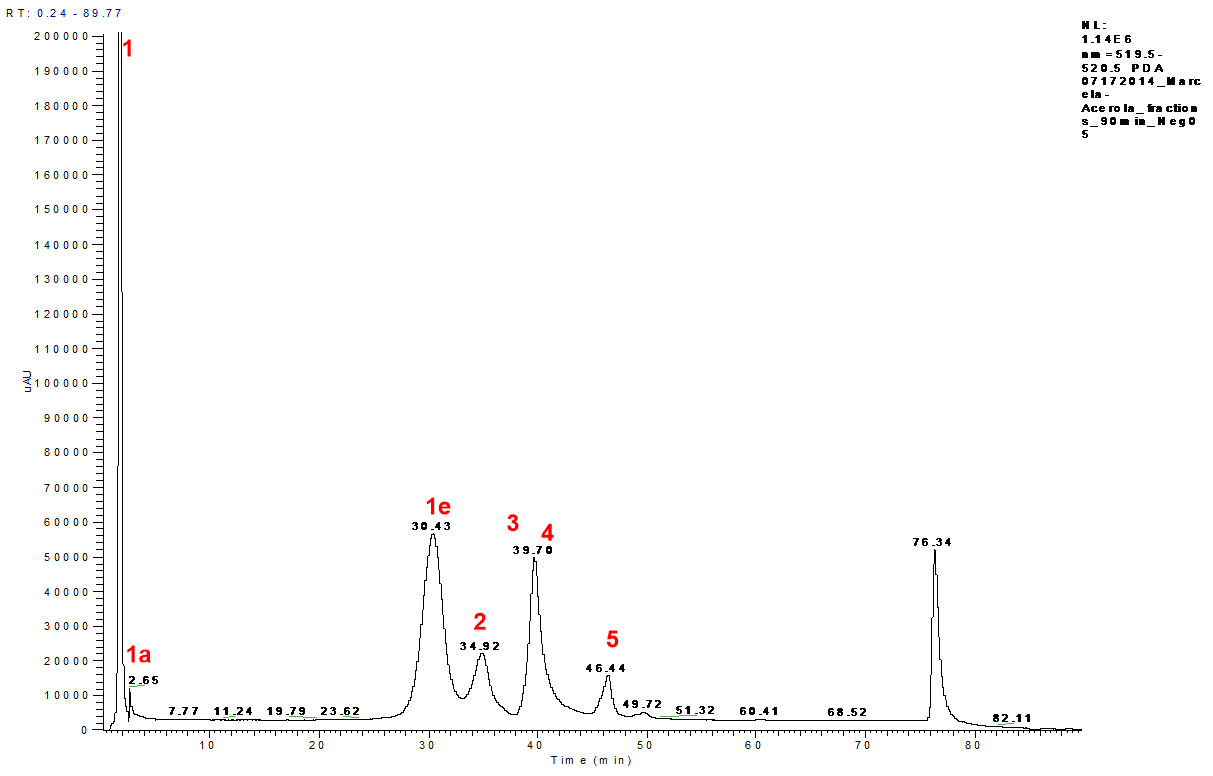


**UV- (Day7, Fraction F4)**


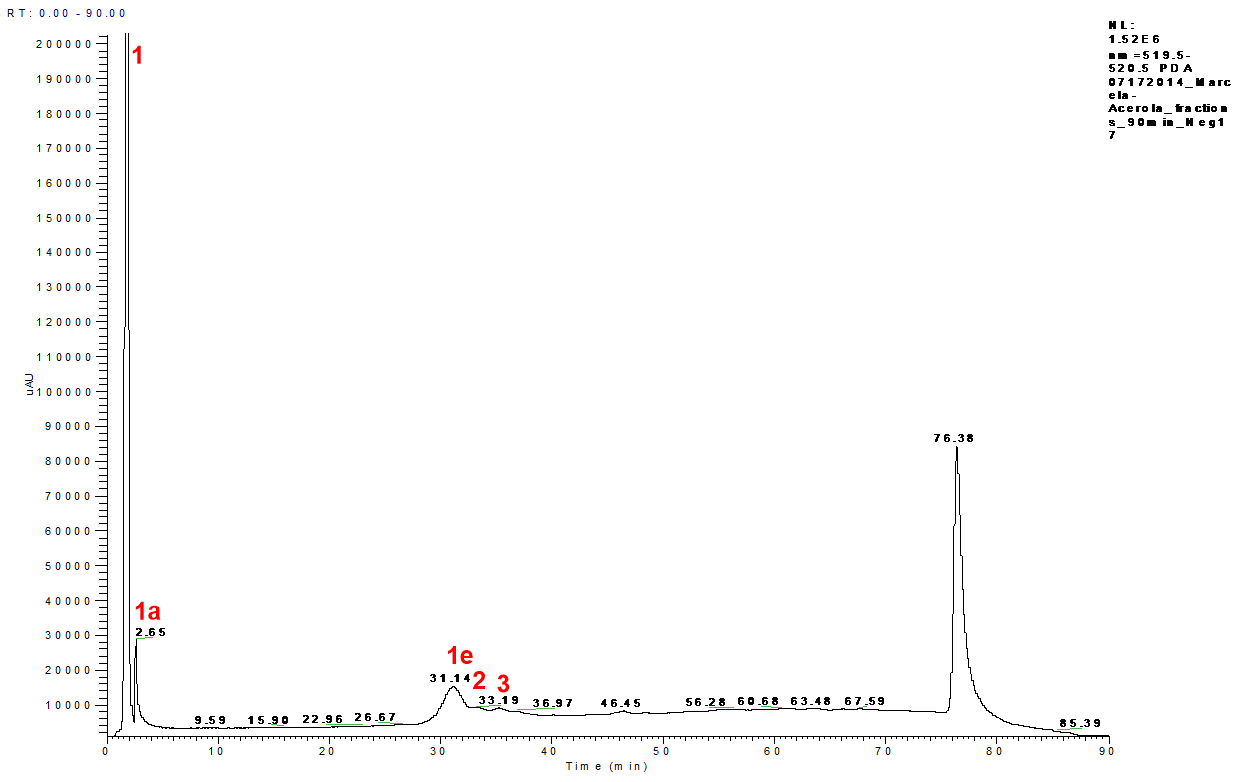


**UV+ (Day7, Fraction F4)**


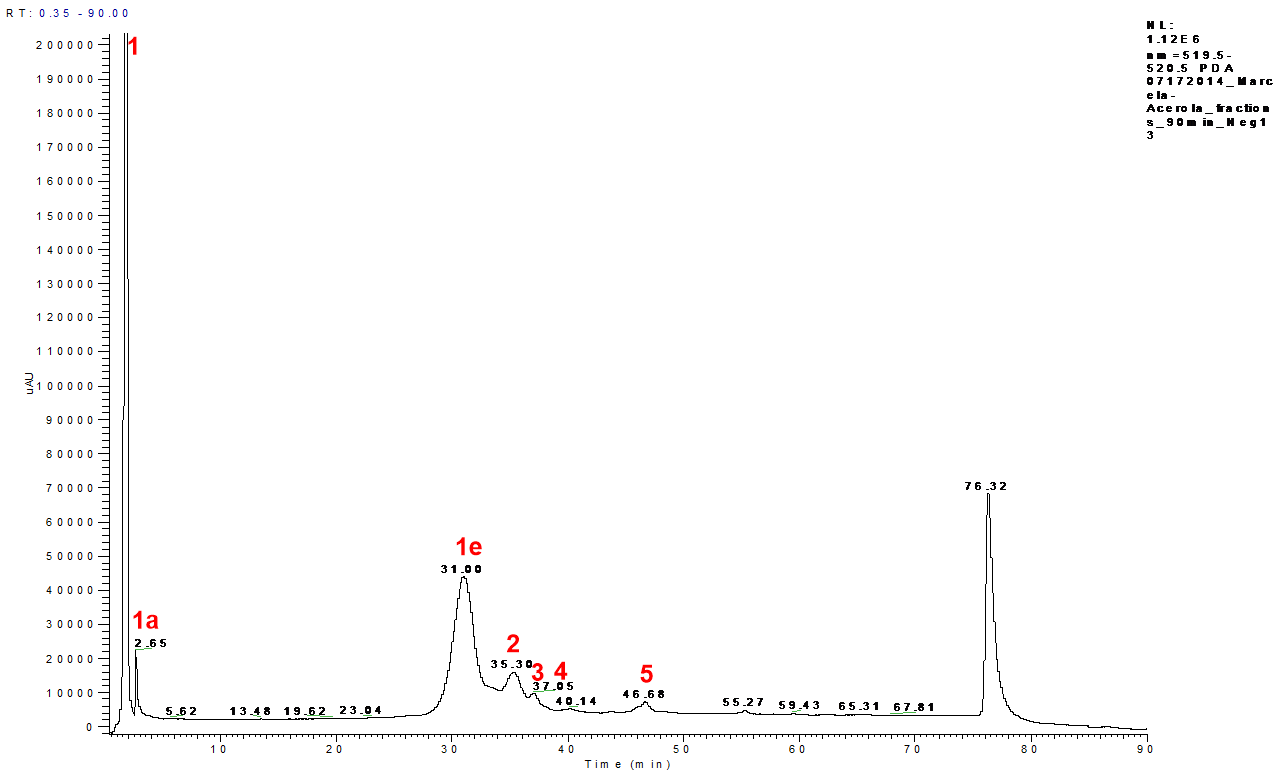


**Identification of phenolic compounds in acerola Fractions F3 and F4 by LCMS**

Peak **1** at retention time in minute (RT) 1.82-1.84 gave [M-H]- *m/z* 175 and gave fragment peak at *m/z* 136.01 and this compound was identified as ascorbic acid and peak **1a** at RT, 2.62 gave [M-H]- m/z 173 and also m/z [M-H]^-^175 gave fragmentation peak at m/z 135.9 similar to ascorbic acid and a peak at 115.2, hence this compound was identified as dehydroascorbic acid. As ascorbic acid and dehydroascorbic acids are interconvertible it is likely to see both the masses together. Vitamin C is widely found in acerola fruit and is a rich source of vitamin C (*2*). Ascorbic acids and dehydroascorbic acids have been previously investigated in acerola fruit (*3*). Peak **1b** at RT 3.12-3.42 gave [M-H]^-^ *m/z* 159 [M-175-16]^-^ indicating a hydroxyl group elimination from the ascorbic acid moiety and also gave fragments at *m/z* 137 and 126 similar to ascorbic and dehydroascorbic acids, suggesting this product could be associated to degradation of vitamin C. Hence this compound was tentatively identified as deoxy ascorbic acid. We did not find any reports for deoxy ascorbic acids but degradation of vitamin C has been well studied and different byproducts have been obtained, however, some of them remain unidentified (*4*) and hence further work is needed to determine if deoxy ascorbic acid could be one of them.

Peak **1c** at RT 19.5-20.5 gave [M-H]^-^ *m/z* 169 and fragment at *m/z* 126.5 and hence this compound was identified as gallic acid. Peak **1d** at RT 21.3-22.5 gave [M-H]^-^ *m/z* 337 and gave fragments at *m/z* 191, 173 and 163 and hence this compound was identified as coumaroyl quinic acid. Similar fragmentation pattern for coumaroyl quinic acid was observed in the Amazonian fruit *Caryocar villosum* (*5*).

Compound **1e** at RT 30.25 and compound **2** at RT 33.03 gave molecular ions at m/z 433 and m/z 417 each and by MS/MS experiment a major fragment at *m/z* 270 and *m/z* 287, respectively, was obtained hence these compounds were identified as cyanidin-3-rhamnoside and pelargonidin 3-rhamnoside respectively (*6*) Compound **3** at RT 37.37 gave m/z 271 and was identified as pelargonidin, while compound **4** at RT 41.32 gave m/z 451 and yielded MS/MS fragments at m/z 303 suggesting a peonidin derivative and another fragment at m/z 316 and the compound was identified as peonidin-3-xylopyaranoside. Compound **5** at RT 46.19 gave m/z at 595 and yielded a major MS/MS fragment at m/z 287 [M-162-146] indicating a cyanidin derivative and another fragment at m/z 449[M-142], hence this gave an indication of a sugar side chain as rutinoside and hence the compound was identified as cyanidin 3-rutinoside. Some of these anthocyanins were also identified in blue berries of *Vaccinium padifolium* (*7*).

The identification of flavonoids was achieved by comparison of MS fragmentation pattern, UV spectra (nm) and retention time (RT) with data reported in the literature. The MS/MS data mining process began with the identification of the aglycone fragment in order to determinate the flavonoid backbone of each compound, thus quercetin ([M+H] m/z 303 uma), isorhamnetin ([M+H] m/z 317 uma), kaempferol ([M+H] m/z 287 uma) and mainly myricetin ([M+H] m/z 317 uma) were found (*8*).

The MS/MS fragmentation pattern of compounds **6** at RT 47.04, **10** at RT 60.04 and **11** at RT 65.88 showed a fragment at m/z 287, allowing tentatively identifying these compounds as kaempferol *O*-glycosides. The compound **6**, with the precursor ion at m/z 727 [(M+H)]^+^, showed similar fragments at m/z 287 [(M+H)-146-162]^+^ and m/z 581 [(M+H)-146]^+^, this tri-glycoside was identified as kaempferol 3-*O*-robinobioside-7-*O*-arabinofuranoside (*8*). Compound **10** was identified as kaempferol 7-O-neohesperidoside, due to similar MS fragments at *m/z* 287 [(M+H)-146-162]^+^ and *m/z* 258 [(M+H)-146-162-28]^+^ and previously characterized by LC-ESI-MS analysis in cocoa (*Theobroma cacao*)*^5^*. The compound **11** with precursor ion at *m/z* 419, yielded a fragment at *m/z* 287 [(M+H)-132]^+^ and *m/z* 258 [(M+H)-132-28]^+^. This compound, a mono-glycoside, was identified as kaempferol 3-O-arabinofuranoside (*9*).

Compounds **7** at RT 53.18 and **8** at RT 56.06 showed the same precursor ion at *m/z* 479 [(M+H)]^+^ but different RT, and yielded similar fragments at *m/z* 317 [(M+H)-146-162]^+^, allowing tentatively identifying these compounds as isorhamnetin *O*-glycosides. These glycosides were identified as isomers. These compounds, **7** and **8**, were identified as isorhamnetin 3-O-galactoside and isorhamnetin 3-O-glucoside respectively (*10*).

The MS fragmentation analysis of compound **12** at RT 67.19 gave precursor ion at m/z 465, yielded fragments at m/z 303 [(M+H)-162]^+^ and m/z 180 [(M+H)-123]^-^. This compound was identified as quercetin 3-*O*-glucoside (*11*)

Peak **9** at RT 57.16 gave [M-H]^-^ *m/z* 315 and gave fragment at *m/z* 301 [M-H-17]^-^ produced by the elimination of a hydroxyl group hence this compound was identified as isorhamnetin. Peak **13** at RT 67.19 gave [M-H]^-^ *m/z* 599, gave a major fragment ion at *m/z* 301 [M-H-271-17]^-^ indicating the loss of galloyl and hydroxyl moieties (and minor fragments at m/z 328, 431) and hence this compound was identified as Quercitrin-2´´-O-gallate. Quercitrin-2’’-O-gallate was also observed in the seasoning spice *Rhus coriaria* L. (Anacardiaceae), which is a wild edible plant growing in the Mediterranean region (*12*). Compounds **14**-**16** gave major MS^2^ fragment at *m/z* 317 indicating all these compounds were myricetin derivatives. Compounds **14** at RT 73.88 gave [M-H]^-^ *m/z* 495, **15** at RT 74.36 gave [M-H]^-^ *m/z* 479 and **16** at RT 76-77.5 gave [M-H]^-^ *m/z* 465 and hence these compounds were identified as myricetin-3-O-glucuronide, myricetin-3-O-glucoside and myricetin rhamnoside respectively. Recent reports showed that myricetin derivatives were observed in Brazilian tropical fruits and acerola by UPLCMS/MS (*13*).

**Supplementary Table S1.** Average areas of the peaks observed for non UVC and UVC treated acerola fruit at day 0 and day 7

| **Peak** | **Retention time (min)** | **Identification** | **DAY 0** | | | | **DAY 7** | | | |
| --- | --- | --- | --- | --- | --- | --- | --- | --- | --- | --- |
|  |  |  | **UV-** | | **UV+** | | **UV-** | | **UV+** | |
| 1c | 19.5-20.5 | Gallic acid | 105638 | a | 48269 | d | 84285 | b | 53865 | c |
| 1d | 21.3-22.5 | Coumaroyl quinic acid | 86721 | c | 115079 | b | 84575 | c | 174172 | a |
| 1e | 30.25 | Cyanidin 3-rhamnoside | 17286978 | a | 6243927 | b | 698323 | d | 4134936 | c |
| 2 | 33.03 | Pelargonidin 3-rhamnoside | 2332316 | a | 902575 | b | 374961 | c | 784188 | b |
| 3 | 37.37 | Pelargonidin | 61666 | b | 98783 | a | 5558 | d | 24915 | c |
| 4 | 41.32 | Peonidin-3-xylopyranoside | 426968 | b | 2341411 | a | ND |  | 91580 | c |
| 5 | 46.19 | Cyanidin 3-rutinoside | 179295 | c | 1006626 | a | ND |  | 285322 | b |
| 6 | 47.04 | Kaempferol 3-*O*-robinobioside-7-*O*-arabinofurano-side | 428374 | c | 210117 | d | 789744 | a | 435327 | b |
| 7 | 53.18 | Isorhamnetin 3-*O*-galactoside | 639812 | a | 640169 | a | ND |  | 582351 | b |
| 8 | 56.06 | Isorhamnetin 3-O-glucoside | 3588287 | d | 4388613 | c | 7853935 | b | 9197524 | a |
| 9 | 57.16 | Isorhamnetin | 258166 | b | 247700 | c | 290489 | a | ND |  |
| 10 | 60.4 | Kaempferol 7-*O*-neohesperidoside | 738033 | b | 482146 | c | 444416 | d | 996950 | a |
| 11 | 65.88 | Kaempferol 3-*O*-arabinofuranoside | 1505010 | d | 1972178 | c | 2516111 | b | 2587202 | a |
| 12 | 67.19 | Quercetin 3-*O*-glucoside | 604462 | b | 614788 | b | 121475 | c | 1018854 | a |
| 13 | 70.38 | Quercitrin-2”-*O*-gallate | 248505 | b | 182520 | d | 415423 | a | 223251 | c |
| 14 | 73.88 | Myricetin-3-*O*-glucuronide | 4413 | b | 408898 | a | ND |  | ND |  |
| 15 | 74.36 | Myricetin-3-*O*-glucoside | 454517 | b | 4074 | c | 7112 | c | 715824 | a |
| 16 | 76-77.85 | Myricetin rhamnoside | 3063529 | c | 2111942 | d | 5579563 | a | 4765423 | b |

Values represent the mean of three replicates. Values with different letters within the same compound represent statistical difference (p < 0.05) by the Tukey HSD test. Areas of peaks 1e to 5 were quantified at 520 nm (Fraction F4) and areas of peaks 1c, 1d, 6-16 were quantified at 330 nm (Fraction F3). N.D. = Not detected.

**Supplementary Table S2.** Sequences of primers used in gene expression studies.

| Gene identification (Accession number) | Sequence for forward (F) and reverse (R) primers (5’ 🡪 3’) |
| --- | --- |
| GDP-D-mannose pyrophosphorylase (*MgGMP,* DQ229168) | F: TTGTCGAGTCGGGAGTTAGA |
|  | R: CAACCGATGATGCTGCTAGATA |
| GDP-mannose 3’,5’-epimerase (*MgGME,*DQ229167) | F: CCTCTAGTGCTTGCATCTATCC |
|  | R: CTAGCTTCTCCAAGCCGTAAG |
| GDP-L-galactose phosphorylase (*MgGGP,* EU683446) | F: AGGCTTCTTGCAGAGGTTTC |
|  | R: CTCACTGCTATTGCCACTGTAT |
| L-galactose dehydrogenase (*MgGDH,* EU683447) | F: GAACTGAAGGCTGCATGTAAAG |
|  | R: CCAGCACGGTTGGTATATCTT |
| L-galactono-1,4-lactone dehydrogenase *(MgGalDH,* EU683445) | F: GAGCATGCAGGACCTTGATTA |
|  | R: GCTGTCCACCGTTGTTCTAT |
|  |  |

**References:**

1. Nair, V., Bang, W. Y., Schreckinger, E., Andarwulan, N. & Cisneros-Zevallos, L. The protective role of ternatin anthocyanins and quercetin glycosides from butterfly pea (*Clitoria ternatea* Leguminosae) blue flower petals against LPS-induced inflammation in macrophage cells. *J Agric. Food Chem.* 63(28), 6355-6365 (2015).

2. Vendramini, A. L. & Trugo, L. C. Chemical composition of acerola fruit (*Malpighia punicifolia* L.) at three stages of maturity. *Food Chem.* 71(2), 195-198 (2000).

3. Mezadri, T., Villaño, D., Fernández-Pachón, M., García-Parrilla, M. & Troncoso, A. Antioxidant compounds and antioxidant activity in acerola (*Malpighia emarginata* DC.) fruits and derivatives. *J. Food Compos. Anal.* 21(4), 282-290 (2008).

4. Yuan, J.-P. & Chen, F. Degradation of ascorbic acid in aqueous solution. *J. Agric. Food Chem.* 46(12), 5078-5082 (1998).

5. Chiste, R.C., & Mercadante, A.Z. Identification and quantification, by HPLC-DAD-MS/MS, of carotenoids and phenolic compounds from the Amazonian fruit Caryocar villosum. J. Agric. Food Chem. 60(23), 5884-5892 (2012).

6. de Rosso, V.V., Hillebrand, S., Montilla, E.C., Bobbio, F.O., Winterhalter, P. & Mercadante, A.Z. Determination of anthocyanins from acerola (*Malpighia emarginata* DC.) and açai (*Euterpe oleracea* Mart.) by HPLC–PDA–MS/MS. *J. Food Compos. Anal.* 21, 291-299 (2008).

7. Cabrita, L. & Andersen, Ø. M. Anthocyanins in blue berries of *Vaccinium padifolium*. *Phytochemistry* 52, 1693-1696 (1999).

8. Astello-García, M.G., Cervantes, I., Nair, V., del Socorro Santos-Díaz, M., Reyes-Agüero, A., Guéraud, F., Negre-Salvayre, A., Rossignol, M., Cisneros-Zevallos, L. & de la Rosa, A. P. B. Chemical composition and phenolic compounds profile of cladodes from Opuntia spp. cultivars with different domestication gradient. *J. Food Compos. Anal.* 43, 119-130 (2015).

9. Lhuillier, A., Fabre, N., Moyano, F., Martins, N., Claparols, C., Fourasté, I., Moulis, C. Comparison of flavonoid profiles of *Agauria salicifolia* (Ericaceae) by liquid chromatography-UV diode array detection–electrospray ionisation mass spectrometry. *J. Chromatogr. A*, 1160, 13-20 (2007).

10.De Leo, M., De Abreu, M. B., Pawlowska, A., Cioni, P. & Braca, A. Profiling the chemical content of *Opuntia ficus-indica* flowers by HPLC–PDA-ESI-MS and GC/EIMS analyses. *Phytochem. Lett.* 3, 48-52 (2010).

11.Sánchez‐Rabaneda, F., Jáuregui, O., Casals, I., Andrés‐Lacueva, C., Izquierdo‐Pulido, M. & Lamuela‐Raventós, R.M. Liquid chromatographic/electrospray ionization tandem mass spectrometric study of the phenolic composition of cocoa (*Theobroma cacao*). *J. Mass Spectrom.* 38, 35-42 (2003).

12. Abu-Reidah, I.M., Ali-Shtayeh, M.S., Jamous, R. M., Arráez-Román, D. & Segura-Carretero, A. HPLC–DAD–ESI-MS/MS screening of bioactive components from Rhus coriaria L.(Sumac) fruits. *Food Chem.* 166, 179-191 (2015).

13. Bataglion, G.A. da Silva, F.M. Eberlin, M.N. & Koolen, H.H. Determination of the phenolic composition from Brazilian tropical fruits by UHPLC–MS/MS. *Food Chem.* 180, 280-287 (2015).
